# TcCARP3 modulates compartmentalized cAMP signals involved in osmoregulation, infection of mammalian cells, and colonization of the triatomine vector in the human pathogen *Trypanosoma cruzi*

**DOI:** 10.1128/mbio.00994-25

**Published:** 2025-05-23

**Authors:** Joshua Carlson, Milad Ahmed, Riley Hunter, Syeda Farjana Hoque, Joshua B. Benoit, Miguel A. Chiurillo, Noelia Lander

**Affiliations:** 1 Department of Biological Sciences, University of Cincinnati 2514, Cincinnati, Ohio, USA; University of Wisconsin-Madison, Madison, Wisconsin, USA

**Keywords:** adenylate cyclase, Chagas disease, contractile vacuole complex, flagellar distal domain, kissing bugs, metacyclogenesis, regulatory volume decrease, trypanosomes

## Abstract

*Trypanosoma cruzi* is the causative agent of Chagas disease, a zoonotic infectious disease considered a leading cause of cardiomyopathy, disability, and premature death in the Americas. This parasite spends its life between a mammalian host and an arthropod vector, undergoing essential transitions among different developmental forms. How *T. cruzi* senses microenvironmental changes that trigger cellular responses necessary for parasite survival has remained largely unknown. Cyclic AMP (cAMP) is a universal second messenger that has been shown to regulate key cellular processes in trypanosomes, in which cAMP response proteins (CARPs) have been proposed to be modulators or effectors of a PKA-independent signaling pathway. In this study, we aimed to investigate the role of TcCARP3 in cAMP signaling throughout the *T. cruzi* life cycle. Our results show that TcCARP3 shares a dual localization (flagellar tip and contractile vacuole complex) with adenylate cyclase 1 (TcAC1) in the main developmental stages of the parasite. We also found that TcCARP3 directly interacts with several TcACs, modulating the intracellular content of cAMP. Through generation of *TcCARP3* knockout, addback, and overexpression cell lines, we showed that modulation of gene expression affects the parasite’s ability to differentiate, respond to osmotic stress, invade mammalian cells and replicate within them, and colonize the hindgut of the triatomine vector. In addition, we identified several signaling proteins interacting with TcCARP3 in what we propose are cAMP signaling microdomains. Our results unveil a key role for TcCARP3 as a modulator of cAMP signals necessary for parasite differentiation and survival throughout the *T. cruzi* life cycle.

**IMPORTANCE:**

Cyclic AMP (cAMP) signaling pathways are poorly understood in the stercorarian parasite *Trypanosoma cruzi*. Specifically, the mechanisms driving the activation of TcACs in response to microenvironmental stress are completely unknown. This study unveils the role of TcCARP3 in modulating the content of cAMP through the interaction with several TcACs and putative cAMP effectors in *T. cruzi*. Particularly, TcCARP3 interacts with TcAC1 in the main developmental stages of this parasite’s life cycle, where both proteins display a dual localization pattern. These results provide new evidence supporting the compartmentalization of cAMP signals in trypanosomes. Moreover, our data unequivocally demonstrates that TcCARP3 is required for essential cellular processes, such as response to osmotic stress, host cell invasion, intracellular replication, and the ability to colonize the hindgut of the triatomine vector. In summary, we found that TcCARP3 is an adenylate cyclase interactor that modulates cAMP signals necessary for the life cycle progression of *T. cruzi*.

## INTRODUCTION

*Trypanosoma cruzi* is the protozoan parasite that causes Chagas disease, one of the neglected tropical diseases of greatest public health importance in the Americas. According to the most recent data available from the World Health Organization, an estimate of 7 million people are affected by this debilitating disease, with 70 million people at risk of infection (1). Many people with Chagas disease are not even aware of their condition*,* as initially the disease shows non-specific flu-like symptoms, and patients resume normal daily life, but the infection persists. It is not until decades later that roughly a third of patients will develop serious health conditions such as cardiac, gastrointestinal, or neurological complications that can result in death (2, 3). Currently, only two drugs are available to treat Chagas disease while patients are in the acute phase of infection, but once it progresses to the chronic phase, these drugs become ineffective (4). While endemic to 21 Latin American countries, an increasing number of Chagas disease cases have been reported in many non-endemic regions including the United States, Canada, Europe, the Middle East, Asia, and Australia (3). The main driving forces behind this spread in recent decades are global human migrations and vector colonization of non-rural areas (1, 4). As Chagas disease becomes a global health problem, the development of alternative and more efficient strategies to diagnose and treat *T. cruzi* infections is urgently needed. Understanding this parasite’s biology is crucial for the rational development of new antiparasitic interventions.

*T. cruzi* is a stercorarian parasite with a digenetic life cycle alternating between an arthropod vector (a triatomine bug) and a mammalian host. The natural transmission of *T. cruzi* to humans occurs through contact with the feces of infected triatomine bugs, commonly known as kissing bugs. Infective metacyclic trypomastigotes (MTs) are present in the urine and feces of the vector and are transmitted to the mammalian host via skin wound or mucous membranes. Once inside the organism, MTs can invade any nucleated cell and differentiate into the intracellular amastigote. After several rounds of replication, amastigotes differentiate into infective cell-derived trypomastigotes that are eventually released into the bloodstream. These trypomastigotes can then invade other host cells or can be taken up by another triatomine bug. Once inside the vector, trypomastigotes differentiate into the proliferative epimastigotes in the midgut. Over the course of several weeks, epimastigotes migrate to the hindgut of the triatomine bug, where they differentiate into infective MTs, in a process known as metacyclogenesis (reviewed by reference 5). Throughout its life cycle, *T. cruzi* encounters significant microenvironmental changes, including drastic fluctuations in temperature, pH, nutrient availability and composition, and osmolarity (reviewed by references 6, 7). The underlying mechanisms of how *T. cruzi* senses these environmental changes and triggers specific cellular responses that drive developmental transitions are still poorly understood.

As in many eukaryotic organisms, *T. cruzi* relies on cAMP signaling to mediate cellular responses to external stimuli (7, 8). However, to date, this pathway has been better characterized in the mammalian system, where the nature and function of specific proteins determining the spatiotemporal dynamics of the signals have been well described (9, 10). Canonically, an external stimulus is received by a G protein-coupled receptor (GPCR), and that signal is transduced to adenylate cyclases (ACs), enzymes responsible for the catalytic conversion of ATP into cyclic AMP (cAMP) (11). This second messenger interacts with effector proteins such as protein kinase A (PKA), exchange protein activated by cAMP (EPAC), or cyclic nucleotide-gated ion channels (CNGCs). The signal is then abolished with the degradation of cAMP into AMP by phosphodiesterases (PDEs) (12). In trypanosomes, catalytically active ACs and PDEs have been well characterized (13–20), but the canonical effectors of cAMP are either absent or not responsive to cAMP in these parasites (21, 22). Furthermore, genes encoding GPCRs are absent in the genomes of trypanosomatids (23, 24), leading to fundamental questions such as what mechanism drives the activation of ACs in these parasites, and what downstream effectors are modulated by cAMP upon its synthesis (25–27). In *T. cruzi*, ACs comprise a multigene family that we have classified into five groups of putative receptor-type ACs (AC I–V) (17). In this parasite, cAMP signaling has been specifically linked to the cellular processes of cell adhesion (17), metacyclogenesis (17, 28–32), and response to osmotic stress (16, 17, 33–36), but further research is needed to understand the molecular mechanisms driving these responses. A promising group of proteins that includes putative cAMP effectors in trypanosomatids is known as cAMP response proteins (CARPs). CARPs are kinetoplastid-specific proteins that were first identified in *T. brucei* through genome-wide RNAi screenings for resistance to lethal concentrations of PDE inhibitors (37, 38). CARP1–CARP4 were identified first when screening for genes conferring resistance to CpdA (37). A more recent RNAi screening performed through deep sequencing confirmed CARP1–4 as genes that confer resistance to this PDE inhibitor and identified six other CARPs (CARP6–CARP11) in *T. brucei* (38). Two of these genes contain predicted cyclic nucleotide binding domains (CARP1 and CARP11), and only CARP3 has been described in *T. brucei* as an upstream regulator of cAMP signaling that modulates the activity of ACs (39, 40). CARPs are not structurally related to each other. However, they all play a role in cAMP signaling. CARP3 has been described in *T. brucei* (TbCARP3) as a multi-AC modulator involved in social motility (SoMo) and colonization of the insect vector (39, 40). We recently found that the *T. cruzi* homolog (TcCARP3) shows a peculiar dual localization pattern in the flagellar distal domain (flagellar tip) and the contractile vacuole complex (CVC) of the parasite (17). These two compartments are directly involved in cell adhesion/metacyclogenesis and response to osmotic stress, respectively (5, 33, 35, 36, 41–43). In addition, these processes have previously been linked to cAMP signaling (16, 28, 30–32, 34, 35, 44). Furthermore, TcCARP3 localization mirrored that of the catalytically active TcAC1, and their interaction was demonstrated through immunoprecipitation and mass spectrometry analysis using TcAC1 as bait (17). Considering the localization of TcCARP3 and TcAC1 in these two subcellular compartments, their peculiar presence at the CVC membrane, where TcAC1 has been predicted to interact with TcCARP3 through its cytosolic catalytic domain (39), and the stark differences in the biology of *T. brucei* (salivaria) and *T. cruzi* (stercoraria) parasites, we aimed to investigate the role of TcCARP3 in cAMP signaling throughout the *T. cruzi* life cycle. In this study, we modulated the expression of TcCARP3 through the generation of mutant cell lines and evaluated their phenotype in different developmental stages, *in vitro* and *in vivo*. Our results shed light on the role of TcCARP3 in cAMP signaling as well as its main protein interactors in two distinct subcellular compartments, leading to cellular responses necessary for parasite survival and transmission during the progression of *T. cruzi* life cycle.

## RESULTS

### CARP3 is a *Trypanosoma*-specific protein with dual localization in *T. cruzi*

**Fig 1:**
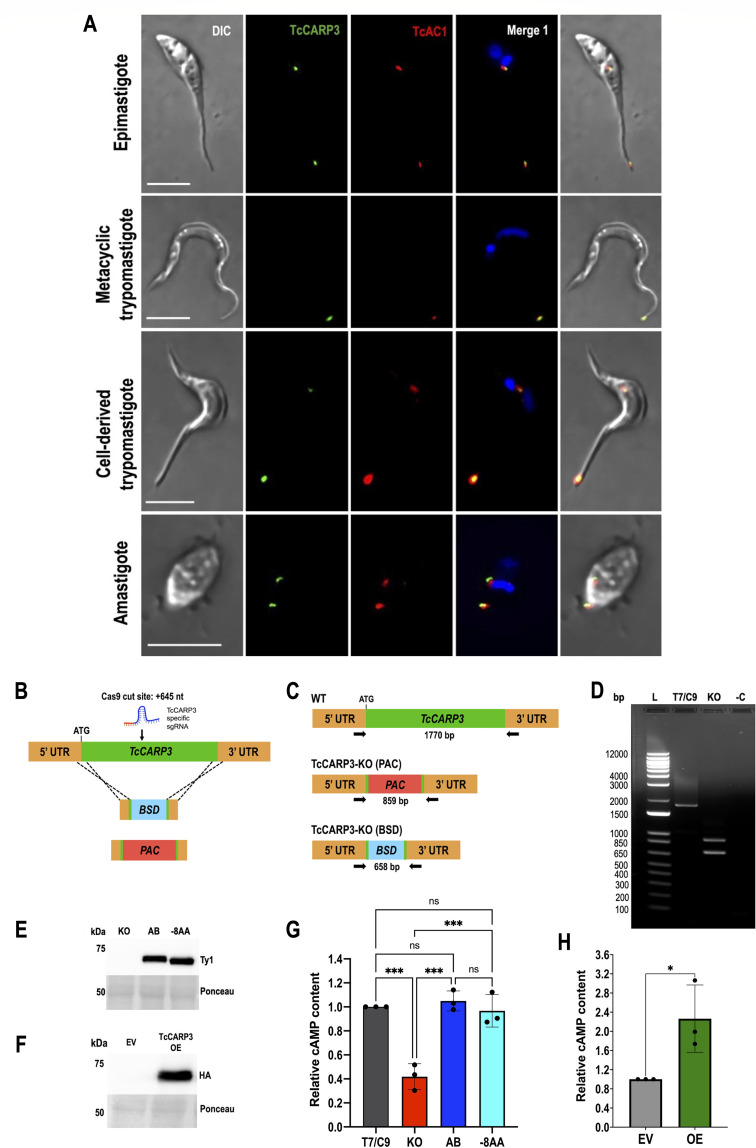
Localization of TcCARP3 and generation of mutant cell lines. (A) IFAs were performed using TcAC1-3xHA/TcCARP3-3xc-Myc dually tagged cell line under hypoosmotic stress in different developmental stages of *T. cruzi*, as shown in labels at the left side of each IFA panel. Images from left to right show DIC, TcCARP3 (green), TcAC1 (red), TcCARP3 and TcAC1 merged (yellow) with DAPI (blue), and with DIC. DAPI was used to stain the nucleus and kinetoplast. Scale bars: 5 μm. (B) Schematic representation of CRISPR/Cas9-mediated knockout strategy for *TcCARP3*. (C) Predicted sizes of PCR products using parental or *TcCARP3*-KO gDNA. (D) PCR products to verify the genotype of TcCARP3 mutants resolved in 1% agarose gel. Lane labels: 1 kb plus ladder (L), T7RNAP/Cas9 (T7/C9), TcCARP3-KO (KO), and negative control (−C). (E) Western blot analysis confirming expression of TcCARP3 in the *TcCARP3*-AB (61.15 kDa) and *TcCARP3*-8AA (61.05 kDa) cell lines. *TcCARP3*-KO was included as a negative control. (F) Western blot analysis confirming expression of TcCARP3-3xHA in the *TcCARP3*-OE cell line. The empty vector (EV) cell line was used as a negative control. Ponceau red staining was used as a loading control in (E) and (F). (G) and (H) Intracellular cAMP content in *TcCARP3* mutant epimastigotes. Statistical analyses performed were one-way ANOVA with Tukey’s multiple comparisons (G) and Student’s *t*-test (H). Values are means ± SD from three independent experiments. *P < 0.05, ***P < 0.001.

*TcCARP3* (TriTrypDB gene ID: TcYC6_0045920) is a 1,548 bp single-copy gene annotated as a hypothetical protein on chromosome 16 of *T. cruzi* Y C6 genome (45, 46). The predicted protein has a molecular weight of 58.16 kDa and is 515 amino acids in length, with a high-confidence predicted post-translational modification (PTM) of myristoylation occurring on amino acid number 2, glycine, immediately following the start methionine (47). TcCARP3 tertiary structure also has a predicted TPR-like tetratricopeptide-like helical domain spanning from amino acids 13–155, as predicted by InterPro (IPR011990) in TriTrypDB (46, 48) (Fig. S1A). This domain has important implications in mediating protein-protein interactions and the assembly of multi-protein complexes in a wide range of proteins from a diverse set of organisms (49–52). *TcCARP3* shares 62.47% nucleotide sequence identity with its ortholog in *T. brucei, TbCARP3* (TriTrypDB gene ID: Tb427.07.5340) and 50.09% identity at the amino acid level, with 68.41% protein similarity. Conversely, *CARP3* orthologs are absent in *Leishmania spp*. We have previously shown that TcCARP3 and TcAC1 (TriTrypDB gene ID: TcYC6_0015740) co-localize in two different compartments of *T. cruzi* epimastigotes under hypoosmotic stress: the flagellar tip and the CVC (17). This dual localization pattern was also observed in epimastigotes under normal (isosmotic) conditions (Fig. S1B). To confirm TcCARP3’s localization in this developmental stage, we endogenously tagged TcCARP3 with a C-terminal 3xTy1 tag as described in Materials and Methods. The expression and dual localization of TcCARP3 was confirmed using this cell line (Fig. S2). Then, using the dually tagged cell line expressing TcCARP3-3xc-Myc and TcAC1-3xHA (17), we analyzed the localization of both proteins by immunofluorescence assay (IFA) in the four main developmental stages of *T. cruzi*. IFAs were done under hypoosmotic conditions to better visualize the central vacuole of the CVC. Our results indicate that these proteins co-localize in all developmental stages (epimastigotes, MTs, amastigotes, and cell-derived trypomastigotes), showing the previously described dual localization pattern in all of them, except in MTs, where both proteins localized to the tip of the flagellum only (Fig. 1A). The flagellar tip localization of TcCARP3 has been previously reported in the intracellular amastigote stage (53), but we also observed it at the CVC of *T. cruzi* epimastigotes (17). Here, we have confirmed this dual localization pattern in the mammalian stages of the parasite.

### Modulation of TcCARP3 expression affects the cAMP content in *T. cruzi* epimastigotes

To analyze the effect of *TcCARP3* gene ablation in different developmental forms, we generated a *TcCARP3* knockout mutant by CRISPR/Cas9 (*TcCARP3*-KO), as described in Materials and Methods (Fig. 1B). After confirming the genotype of this mutant (Fig. 1C and D), clonal populations were obtained by serial dilutions, and the resulting clones were verified by PCR (Fig. S3). The *TcCARP3* gene was then added back by cloning its ORF or a truncated version of it into the pTREXh-2xTy1 expression vector and transfecting a clonal population of *TcCARP3*-KO epimastigotes to generate the full-length *TcCARP3* addback cell line (*TcCARP3*-AB) and a shorter version (*TcCARP3*-8AA), where the predicted myristoylation signal was removed. Expression of TcCARP3-2xTy1 in *TcCARP3*-AB and *TcCARP3*-8AA parasites was verified by western blot analysis (Fig. 1E). A TcCARP3 overexpressing cell line (*TcCARP3*-OE) was also obtained by cloning the ORF of *TcCARP3* into pTREXn-3xHA vector (17). This construct was used to transfect wild-type epimastigotes. Expression of TcCARP3-3xHA in a clonal population was confirmed by western blot analysis (Fig. 1F). Subsequently, we evaluated the levels of cAMP in *TcCARP3* mutant parasites using the luminescent cAMP Glo-Assay (Promega), as described in Materials and Methods. Our results indicate that *TcCARP3*-KO epimastigotes exhibit a significantly lower content of cAMP than that of control cells, and the normal phenotype was restored in the addback cell line (Fig. 1G). Interestingly, the relative levels of cAMP in *TcCARP3*-8AA parasites were similar to those of T7/Cas9 and *TcCARP3*-AB parasites (Fig. 1G). We also observed that parasites overexpressing TcCARP3 have a higher cAMP content than the empty vector (EV) control (Fig. 1H), although the difference was less pronounced than that observed between *TcCARP3*-KO and T7/Cas9 parasites (Fig. 1G and H). These results indicate that TcCARP3 positively modulates the total cAMP content in *T. cruzi* epimastigotes.

### TcCARP3 ablation affects growth and differentiation of *T. cruzi* epimastigotes

**Fig 2:**
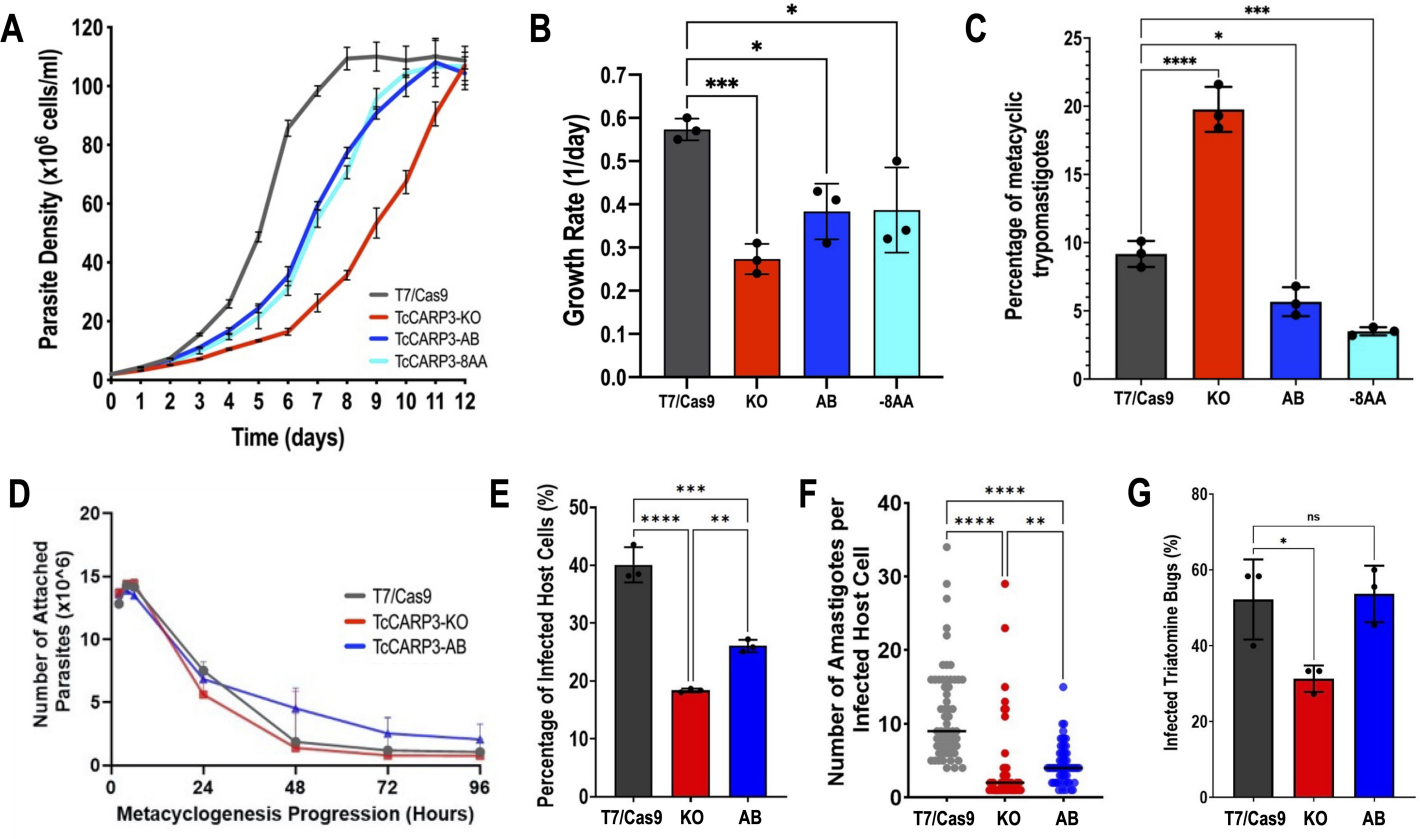
Phenotype of *TcCARP3*-KO and rescued mutants. (A) Growth of T7/Cas9, *TcCARP3*-KO, *TcCARP3*-AB, and *TcCARP3*-8AA epimastigotes in LIT medium. (B) Growth rate was analyzed during the exponential phase of the curve (Days 3–6). (C) Metacyclogenesis *in vitro* of the same mutants. Values are means ± SD, *n* = 3. One-way ANOVA with Dunnett’s multiple comparisons. (D) Adhesion assay with T7/Cas9, *TcCARP3*-KO, and *TcCARP3*-AB parasites. No significant differences were observed at any time point. Values are means ± SD, *n* = 3. Two-way ANOVA with Dunnett’s multiple comparisons. (E) Percentage of infected host cells 24 h post-infection with T7/Cas9, *TcCARP3*-KO, and *TcCARP3*-AB trypomastigotes. Values are means ± SD, *n* = 3. One-way ANOVA with Tukey’s multiple comparisons. (F) Number of intracellular amastigotes per infected host cell 72 h post-infection with T7/Cas9, *TcCARP3*-KO, and *TcCARP3*-AB trypomastigotes. Black line indicates median value per cell line, *n* = 60. Kruskal-Wallis test with Dunn’s multiple comparisons. (G) Percentage of infected triatomine bugs *in vivo*. Kissing bugs were fed blood laden with *T. cruzi* epimastigotes, and hindguts were dissected 30 days later and examined under a microscope for the presence of parasites. Values are mean ± SD, *n* = 3 independent experiments with 9–15 insects per group infected with each specific *T. cruzi* cell line. One-way ANOVA with Tukey’s multiple comparisons. *P < 0.05, **P < 0.01, ***P < 0.001, ****P < 0.0001.

To further explore the phenotype of TcCARP3 mutants, we performed a growth curve using the parental cell line (T7/Cas9) as control, *TcCARP3*-KO, *TcCARP3*-AB, and *TcCARP3*-8AA epimastigotes (Fig. 2A). The growth rate in LIT medium was examined at early exponential phase (days 3–6), resulting in a significantly lower growth of *TcCARP3*-KO parasites compared to control cells, while the normal phenotype was partially rescued in *TcCARP3*-AB and *TcCARP3*-8AA epimastigotes (Fig. 2B). These mutant cell lines were also used to evaluate metacyclogenesis, a differentiation process that is essential for parasite development within the triatomine vector and further transmission to mammalian hosts. *In vitro* metacyclogenesis was performed by incubating epimastigotes in triatomine artificial urine (TAU) to simulate the conditions in the hindgut of the triatomine bug (54). Then, we evaluated the percentage of MTs by fluorescence microscopy upon DAPI staining. Interestingly, *TcCARP3*-KO parasites showed a significantly higher percentage of MTs compared to control cells, and this phenotype was rescued by *TcCARP3*-AB and *TcCARP3*-8AA parasites (Fig. 2C). As *TcCARP3*-8AA epimastigotes exhibited the same cAMP content, growth, and differentiation phenotype as *TcCARP3*-AB parasites, we did not include this mutant in further phenotype analyses. In the kissing bug, metacyclogenesis is preceded by attachment of the parasite through the flagellar tip to the hindgut cuticle (5, 41). To test the ability of *TcCARP3*-KO cells to adhere to the flask surface during metacyclogenesis, we performed an *in vitro* adhesion assay by placing epimastigotes in differentiation media (54) and counting the number of cells in the supernatant at different time points. Surprisingly, we did not observe a significant difference in the adhesion capacity of *TcCARP3*-KO, *TcCARP3*-AB, and control parasites (Fig. 2D), indicating that the metacyclogenesis phenotype observed in these mutants is independent of their adhesion phenotype.

### Ablation of a predicted myristoylation signal does not alter TcCARP3 dual localization

Myristoylation is a PTM that involves the addition of a 14-carbon unsaturated fatty acid chain to a subset of N-terminal glycine residues. This PTM has important implications in membrane association and localization of proteins (55). TcCARP3 exhibits two predicted myristoylation sites, the glycine residues at the second (highest score, in a consensus sequence) and eighth positions in the N-terminal end of the protein (Fig. S4A). To evaluate if this predicted myristoylation signal was required for TcCARP3 dual localization in *T. cruzi,* we transfected *TcCARP3*-KO epimastigotes with a pTREXh-2xTy1 vector containing a truncated version of *TcCARP3* (*TcCARP3*-8AA, Fig. 1E), where the two glycine residues encoded within the first eight amino acids were deleted. These parasites showed the same dual localization pattern (the flagellar tip and CVC) as *TcCARP3*-AB cells (Fig. S4B and C), suggesting that the predicted myristoylation signal of TcCARP3 is not required for its dual localization in *T. cruzi* epimastigotes. A 3D structure of TcCARP3-8AA predicted by AlphaFold shows an intact motif of interaction with ACs at the N-terminal region of this mutant, as described in *T. brucei* (39), which suggests that this modification should not affect the interaction of both proteins (Fig. S4D). The normal localization of TcCARP3-8AA explains the lack of phenotype observed in this mutant (Fig. 1G and 2A through C).

### TcCARP3 is necessary for *T. cruzi* invasion and intracellular replication in mammalian cells

To progress in their life cycle, *T. cruzi* MTs invade mammalian host cells and intracellularly differentiate into replicative amastigotes. We previously found that cAMP modulates the ability of parasites to invade host cells and replicate within them (17). Based on this observation, we evaluated the invasion and intracellular replication phenotype of TcCARP3 mutants using standard methods. Cell-derived trypomastigotes from T7/Cas9, *TcCARP3*-KO, and *TcCARP3*-AB cell lines were used to infect human foreskin fibroblasts (hFFs). These infected cells were fixed and mounted onto slides with DAPI. The number of infected host cells (24 h post-infection) and the number of amastigotes per infected cell (72 h post-infection) were determined by fluorescence microscopy. The percentage of infected host cells was significantly lower when infecting them with *TcCARP3*-KO trypomastigotes than when using control parasites, while *TcCARP3*-AB mutant partially restored the normal phenotype (Fig. 2E). In addition, the intracellular replication of *TcCARP3*-KO amastigotes was also hindered, with infected host cells showing a significantly lower number of amastigotes compared to those infected with T7/Cas9 parasites. Again, this phenotype was partially rescued by the *TcCARP3*-AB mutant (Fig. 2F). Our results indicate that TcCARP3 is required by cell-derived trypomastigotes to invade mammalian cells and by *T. cruzi* amastigotes to replicate within them.

### *TcCARP3* ablation impairs the parasite’s ability to colonize the hindgut of the triatomine vector

To progress into their life cycle and undergo a successful transmission to the mammalian host, *T. cruzi* parasites must establish an efficient infection in the triatomine vector, the kissing bug, and finally reach the hindgut, where replicative epimastigotes differentiate into infective MTs. Our results from metacyclogenesis *in vitro* (Fig. 2C) indicate that TcCARP3 is involved in this differentiation process. To provide further evidence, we assessed the ability of *TcCARP3* mutants to establish an infection in the triatomine bug *Rhodnius prolixus*, as described in Materials and Methods. Our results indicate that *TcCARP3*-KO epimastigotes show a significantly reduced capacity to colonize the hindgut of kissing bugs, as compared to control parasites. Interestingly, *TcCARP3*-KO was able to differentiate into MTs. A mixed population of epimastigotes and MTs was observed in the hindgut of infected kissing bugs, but the number of infected insects was significantly lower when infecting them with *TcCARP3*-KO mutant (Fig. 2G). The normal phenotype was rescued in the *TcCARP3*-AB cell line, indicating that TcCARP3 is necessary for *T. cruzi* to establish an efficient infection in the triatomine vector. Taken together, our results shed light on the importance of TcCARP3 for the progression of *T. cruzi* life cycle *in vivo*.

### TcCARP3 overexpression does not affect growth and differentiation but impairs *T. cruzi* infectivity

**Fig 3:**
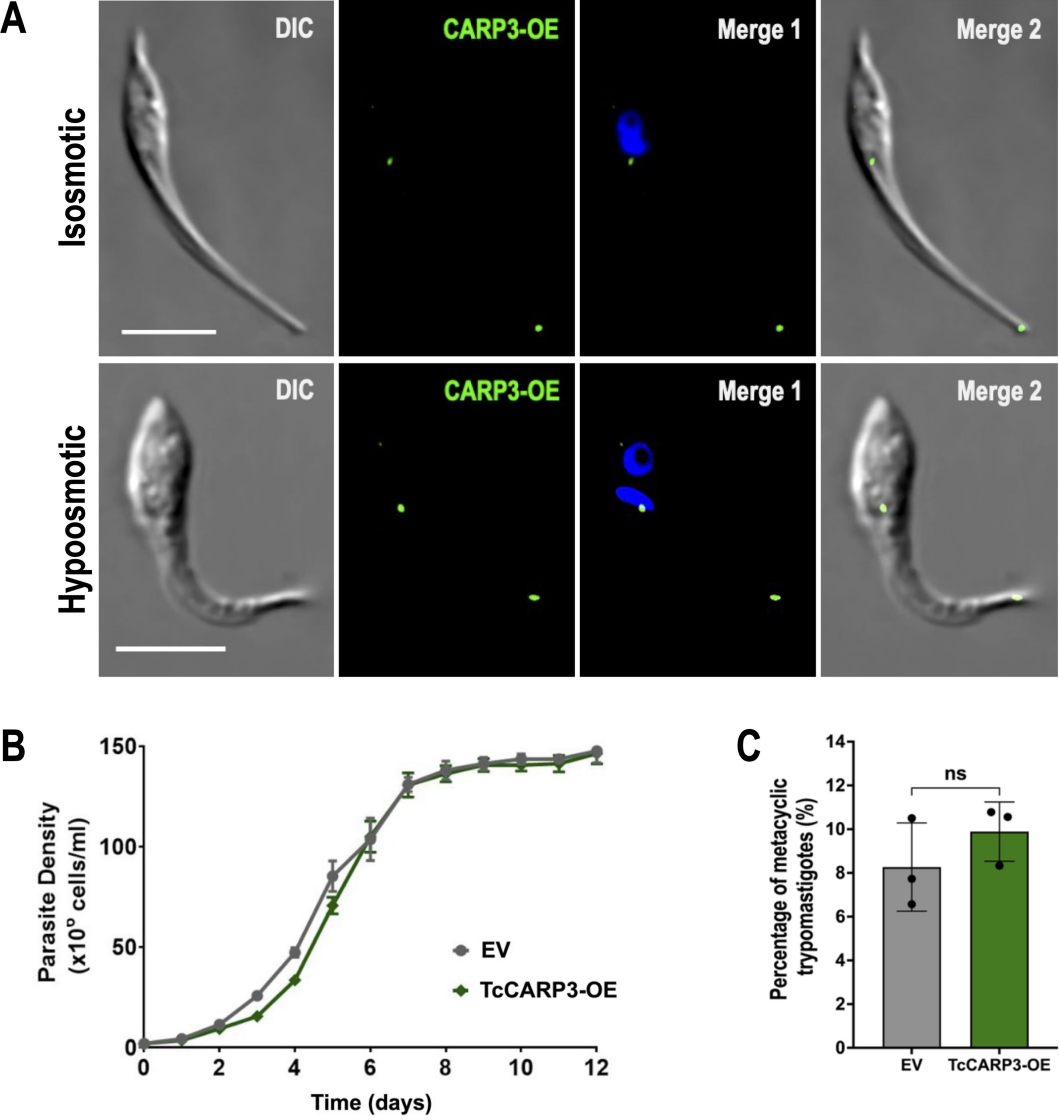
Phenotype of *TcCARP3*-OE epimastigotes. (A) IFAs were performed using the *TcCARP3*-OE cell line under normal (isosmotic) and hypoosmotic conditions. Images from left to right show DIC, TcCARP3-3xHA (CARP3-OE, green), and CARP3-OE merged with DAPI (blue), and with DIC. DAPI was used to stain the nucleus and kinetoplast. Scale bars: 5 μm. (B) Growth of empty vector (EV) and *TcCARP3*-OE epimastigotes in LIT medium. (C) Metacyclogenesis *in vitro* of the same mutants. Values are means ± SD, *n* = 3. Student’s *t*-test.

A mild increase in cAMP content was observed in epimastigotes overexpressing TcCARP3 (Fig. 1H). To further investigate the phenotype of these parasites, we first analyzed the localization of TcCARP3-3xHA overexpressed protein in *T. cruzi* epimastigotes under normal (isosmotic) and hypoosmotic conditions to facilitate the visualization of the CVC. Our IFA results indicate that TcCARP3 overexpression does not affect the dual localization pattern observed in TcCARP3 endogenously tagged epimastigotes in either normal or hypoosmotic conditions (Fig. 3A). We then evaluated the growth of *TcCARP3*-OE epimastigotes in LIT medium, compared to that of the pTREXn-3xHA EV control, and no significant difference was observed (Fig. 3B). We also performed *in vitro* metacyclogenesis for *TcCARP3*-OE and control parasites and did not observe significant differences among them (Fig. 3C). In addition, we infected mammalian host cells with TcCARP3-OE MTs. Interestingly, we were not able to recover enough TcCARP3-OE cell-derived trypomastigotes for use in invasion and replication assays. Host cells infected with this mutant were replete with amastigotes, but their ability to differentiate into trypomastigotes was impaired. After the cells burst, mainly amastigotes were recovered from the culture media, preventing us from performing invasion/replication assays with TcCARP3-OE trypomastigotes. In summary, TcCARP3-OE parasites do not exhibit a metacylogenesis defect, but their ability to establish an efficient infection in hFFs is severely impaired, compared to control cells. This result suggests that even a small change in cAMP content affects *T. cruzi* infectivity.

### TcCARP3 plays a role in the osmoregulatory capacity of *T. cruzi* epimastigotes

**Fig 4:**
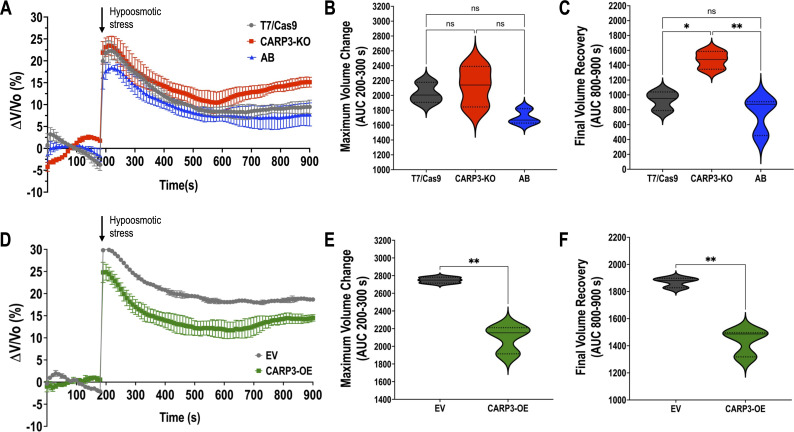
Regulatory volume decrease (RVD) of TcCARP3 mutants under hypoosmotic stress. (A) The light scattering pattern of *T. cruzi* epimastigotes suspended in isosmotic buffer was recorded for 120 s and diluted to a final osmolarity of 115 mOsm/L under constant ionic conditions. Relative changes in cell volume were monitored by measuring absorbance at 550 nm over time in T7/Cas9, *TcCARP3*-KO, and *TcCARP3*-AB parasites. The absorbance values were normalized to the initial volume under isosmotic conditions and expressed as a percentage of volume change. (B) Analysis of the maximum volume change under hypoosmotic conditions. The area under the curve (AUC) in (A) was calculated between 200 and 300 s for all cell lines. (C) Final volume recovery calculated as the AUC in (A) between 800 and 900 s. Values are mean ± SD; *n* = 3; *P < 0.05; **P < 0.01; ns, not significant differences with respect to control cells (one-way ANOVA with Tukey’s multiple comparison). (D–F) Same experiments as in (A–C), but using cell lines empty vector (EV) and *TcCARP3*-OE. Values are mean ± SD; *n* = 3. **P < 0.01 (Student’s *t*-test).

The role of cAMP in the ability of *T. cruzi* epimastigotes to respond to hypoosmotic stress through a process called regulatory volume decrease (RVD) has been previously reported (21, 35–37). Since TcCARP3 co-localizes with TcAC1 in the CVC, an organelle specialized in osmoregulation (33, 43), and differences in total cAMP content were observed in *TcCARP3* mutants, we next evaluated RVD in these parasites, as described in Materials and Methods*. TcCARP3*-KO, *TcCARP3*-AB, and T7/Cas9 epimastigotes were exposed to hypoosmotic stress, and the area under the curve (AUC) in different sections of the light scattering pattern was quantified to determine the maximum volume change (AUC at 200–300 s) and the final volume recovery (AUC at 800–900 s) of the cells (Fig. 4A through C). Our results indicate that *TcCARP3*-KO parasites have a defect in their osmoregulatory capacity, as their final volume recovery was significantly higher than that of control and *TcCARP3*-AB parasites, which restored the normal phenotype of the T7/Cas9 cells (Fig. 4C). Concomitantly, *TcCARP3*-OE parasites showed a more efficient RVD profile compared to that of the EV control, exhibiting a lower maximum volume change and a more efficient final volume recovery in response to hypoosmotic stress (Fig. 4D through F). These results indicate that TcCARP3 plays an important role in the osmoregulatory capacity of *T. cruzi* epimastigotes in response to hypoosmotic conditions.

### Analysis of TcCARP3 protein interactors

**Fig 5:**
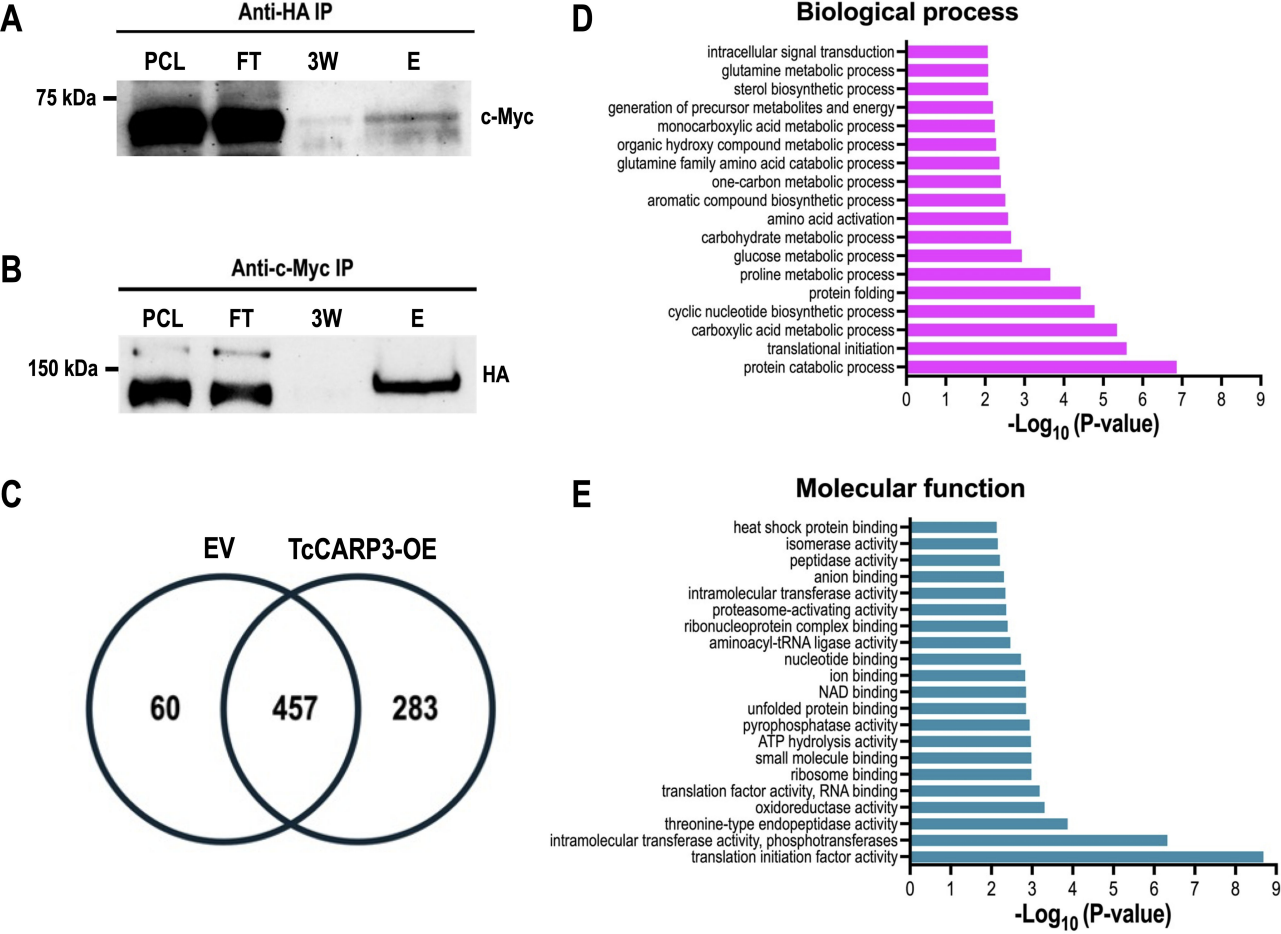
Co-IP of epitope-tagged TcAC1 and TcCARP3 and analysis of the TcCARP3 interactome in epimastigotes. Lanes from left to right are pre-cleared lysate (PCL), flow through (FT), third wash (3W), and eluate (E). (A) Immunoprecipitation with HA beads to capture TcAC1 as bait followed by anti-c-Myc western blot analysis to detect TcCARP3 as prey (64.4 kDa). (B) Immunoprecipitation with c-Myc beads to capture TcCARP3 as bait followed by anti-HA western blot analysis to detect TcAC1 as prey (144 kDa). The upper band seen in the PCL and FT corresponds to Cas9-HA (>150 kDa). (C) Venn diagram showing the number of proteins found in pTREXn-3xHA empty vector (EV) eluates only (60 proteins), the number of proteins found in TcCARP3-OE eluates only (283 proteins), and the number of proteins that were found in both EV and TcCARP3-OE eluates (457 proteins). (D and E) Gene ontology (GO) enrichment analysis for biological process and molecular function of TcCARP3 interacting partners that were absent in the EV control (*P* < 0.05).

The co-localization of TcCARP3 and TcAC1 suggested that these proteins interact in different developmental stages of *T. cruzi* life cycle, as previously observed in *T. cruzi* epimastigotes by mass spectrometry analysis (17). To confirm the interaction between TcCARP3 and TcAC1, we performed a co-immunoprecipitation assay using a dually tagged cell line (TcAC1-3xHA/TcCARP3-3xc-Myc) obtained in our laboratory (17) and HA magnetic beads to trap TcAC1. Different fractions were collected and analyzed by western blot using anti-c-Myc antibodies. We were able to detect TcCARP3 in the eluted fraction (E), and the band was enriched compared to that in the third wash (3W) (Fig. 5A). Then, we co-immunoprecipitated TcCARP3 and TcAC1 using total lysates of the dually tagged cell line and c-Myc magnetic beads to trap TcCARP3. Once again, different fractions were analyzed by western blot, which is now using anti-HA antibodies. Likewise, we were able to detect TcAC1 in the eluate, and the band was clearly enriched compared to the third wash (Fig. 5B).

After confirming the interaction between TcCARP3 and TcAC1, we sought to identify other TcCARP3 protein interactors. For this, we performed an immunoprecipitation assay as described above, using TcCARP3-3xHA as bait protein. In this assay, pTREXn-3xHA EV cell line was used as a control. After immunoprecipitation, snap-frozen eluates were sent to the Mass Spectrometry Core Laboratory at the University of Texas Health Science Center at San Antonio (San Antonio, TX) for mass spectrometry analysis. The group of proteins enriched in the TcCARP3-OE cell line eluates that were absent in the EV control (infinite fold change), with a *P* value <0.05 after Benjamini-Hochberg multiple test correction, and a total spectra count in each TcCARP3-OE replicate ≥1, were deemed as TcCARP3 specific protein interactors (Fig. 5C; Table S1). Interestingly, we found that TcCARP3 interacts with at least seven ACs, from TcAC groups I, III, and V, including TcAC1 (17), and with the regulatory subunit of a PKA-like protein (PKArL), containing two putative cyclic nucleotide binding domains. We also detected several putative proteins involved in cell signaling, such as UNC119, galactokinase-like protein, cAMP-dependent protein kinase catalytic subunit 2, and a homoserine kinase, evidencing the presence of multiple signaling components sharing a subcellular niche with TcCARP3 (Table 1). A gene ontology (GO) enrichment analysis for biological process and molecular function of the 283 TcCARP3 protein interactors is shown in Fig. 5D and E. Taken together, our results suggest that TcCARP3 is a multi-AC regulator that physically interacts with several ACs and with putative cAMP effectors in two cAMP signaling microdomains of *T. cruzi*.

**TABLE 1:** TcCARP3 protein interactors involved in cell signaling

Product description	Gene ID	Representative GO term or domain	Total spectra count	MW (kDa)
Putative receptor-type adenylate cyclase (TcAC3.3)	TcYC6_0073080	Intracellular signal transduction	105/87/118	136
Putative receptor-type adenylate cyclase (TcAC3)	TcYC6_0073060	Intracellular signal transduction	101/88/112	136
Receptor-type adenylate cyclase, putative (TcAC1.1)	TcYC6_0122100	Intracellular signal transduction	38/43/44	141
Receptor-type adenylate cyclase, putative (TcAC1.5)	TcYC6_0051800	Intracellular signal transduction	38/33/45	141
Receptor-type adenylate cyclase, putative (TcAC1.4)	TcYC6_0051770	Intracellular signal transduction	34/30/41	141
UNC119	TcYC6_0076300	GMP PDE delta domain	32/37/39	23
Receptor-type adenylate cyclase, putative (TcAC1)	TcYC6_0015740	Intracellular signal transduction	22/32/30	141
Hypothetical protein, conserved	TcYC6_0066800	Protein kinase-like domain	17/21/22	95
Regulatory subunit of protein kinase A-like protein, putative (PKArL)	TcYC6_0089970	cAMP binding domain	21/20/25	63
Putative receptor-type adenylate cyclase (TcAC5.1)	TcYC6_0051420	Intracellular signal transduction	20/19/29	134
Galactokinase-like protein, putative	TcYC6_0068800	galactokinase domain	13/20/21	52
cAMP-dependent protein kinase catalytic subunit 2 (PKAC2)	TcYC6_0070220	Protein kinase-like domain	7/8/06	38
Homoserine kinase	TcYC6_0098420	Homoserine kinase domain	6/5/07	36

## DISCUSSION

Previous results from our group showed a peculiar localization for TcCARP3 in two putative cAMP signaling microdomains: the CVC and the flagellar tip of *T. cruzi* epimastigotes, where this protein co-localizes with TcAC1 (17). Other cAMP signaling components have been identified in these locations: TcAC4, TcAC5, and TcPDEC2 in the CVC (16, 17), TcAC2 (CVC and flagellar tip) (17), and TcPDEB1 and TcPDEB2 along the flagellum (14, 15), supporting the idea that these two subcellular compartments are indeed cAMP signaling microdomains. We have now confirmed the co-localization of TcCARP3 and receptor-type TcAC1 in three additional developmental stages of *T. cruzi*: MTs, amastigotes, and cell-derived trypomastigotes, where their interaction could be modulating the levels of cAMP in this parasite. Through the generation of *TcCARP3* mutants in which this gene has been either ablated, rescued, or overexpressed, we also demonstrated that TcCARP3 plays a key role in the regulation of cell volume under hypoosmotic stress and in the ability of the parasite to grow and differentiate *in vitro*, invade mammalian cells and replicate within them, as well as to colonize the digestive tract of the triatomine vector. Furthermore, we identified several ACs and other signaling proteins as main interacting partners of TcCARP3, consistent with a role as regulator of compartmentalized cAMP signals in *T. cruzi*. The interaction of CARP3 with several ACs has also been observed in the flagellar tip of the salivarian trypanosome *T. brucei*, where this protein plays a role in SoMo (39). However, here we showed the interaction of TcCARP3 with various TcACs that localize in two different subcellular compartments (flagellar tip and CVC) (17) and explored new cellular processes that are modulated by this protein in *T. cruzi*. Our data highlight the relevance of TcCARP3 as a regulator of compartmentalized cAMP signals throughout the life cycle of *T. cruzi*, a stercorarian trypanosome that is an obligate intracellular parasite.

The visualization of the CVC in *T. cruzi* using conventional microscopy methods is facilitated by exposing the parasites to hypoosmotic stress prior to fixation (17, 35, 42). Under this condition, we observed a dual localization of TcCARP3 in epimastigotes and in the mammalian forms of *T. cruzi*. Interestingly, we found that in MTs, TcCARP3 and TcAC1 co-localize to the flagellar distal domain, but not to the CVC under hypoosmotic conditions. MTs are extremely slender forms with a smaller CVC than other developmental stages (36, 56). TcCARP3 and TcAC1 may not have been detected in the CVC due to their small size in this developmental stage. Another possibility is that these proteins are not present at all at the CVC of these infective forms. The redistribution of TcCARP3 in metacyclic trypomastigotes raises new questions about the role of the CVC in different developmental stages of *T. cruzi*.

TcCARP3 exhibits a high-confidence predicted myristoylation site on the first glycine residue (second amino acid of the protein). Removal of this signal from the N-terminus of the protein did not affect the dual localization of CARP3 to the flagellar tip and the CVC in *T. cruzi* epimastigotes. This result suggests that, unlike what was observed in the *T. brucei* ortholog (39), the predicted myristoylation site of TcCARP3 is either nonfunctional or it is not required for TcCARP3 localization in *T. cruzi*. However, we cannot rule out the presence of non-predicted PTMs in TcCARP3 that could determine its subcellular localization. *Trypanosoma brucei* flagellar member 8 (TbFLAM8) is necessary for TbCARP3 localization to the flagellar tip (39). A similar trafficking mechanism could be directing TcCARP3 to the flagellar tip of *T. cruzi*. However, our mass spectrometry data did not reveal TcFLAM8 as an interacting partner of TcCARP3 in *T. cruzi* epimastigotes. Further research is needed to elucidate the trafficking mechanism of TcCARP3 to these two subcellular compartments.

The flagellar distal domain is a crucial structure for *T. cruzi* attachment and subsequent metacyclogenesis in the hindgut of the triatomine bug (5, 17, 28). We previously observed an increase in cell adhesion during metacyclogenesis when the AC TcAC1 was overexpressed in *T. cruzi* (17). We would then expect increased cAMP local levels in the flagellar tip of *TcCARP3*-KO parasites, in which metacyclogenesis is significantly higher than in control cells. However, due to limitations of the methodology used to measure cAMP content, we were only able to estimate the relative total cAMP content in these parasites. To evaluate the specific cAMP concentration in different subcellular compartments, a biosensor cell line expressing a genetically encoded cAMP indicator should be used, as those available for mammalian cells (10). However, this technology has not been developed in *T. cruzi* due to intrinsic limitations of the model, and so far, we cannot establish a link between local cAMP levels at the flagellar tip and the increased metacyclogenesis observed in *TcCARP3*-KO parasites, as previously reported (17, 28). Attachment of epimastigotes to the lipidic rectal cuticle precedes metacyclogenesis in the kissing bug (reviewed by references 5, 41). Interestingly, here we did not observe an adhesion defect in *TcCARP3*-KO epimastigotes during this differentiation process. Although adhesion is necessary for metacyclogenesis, it is not the only event driving this process. Indeed, there are many biochemical, morphological, and genetic changes that occur during metacyclogenesis (56). Our results suggest that the increased metacyclogenesis observed in *TcCARP3*-KO parasites is not the consequence of a cell adhesion defect. Further research should be performed to elucidate the specific role of TcCARP3 in *T. cruzi* metacyclogenesis. Analysis of gene expression profiles by single-cell RNAseq at different time points during metacyclogenesis of *TcCARP3*-KO parasites could be useful to identify specific altered cellular processes in these mutants.

The second subcellular compartment where TcCARP3 localizes is the CVC. Among other functions, this organelle is involved in the regulation of cell volume under hypoosmotic conditions. In this process, the parasite releases water out of the cell body through an adhesion plaque in the flagellar pocket by pulsatile contractions of the central vacuole (bladder) of the CVC (36). Water efflux follows the tubulin-mediated fusion of acidocalcisomes to the central vacuole and further translocation of the aquaporin (TcAQP) to the CVC in a process known as RVD (33, 35, 43). RVD is a cAMP-mediated process (35, 57) that plays a key role in the survival of *T. cruzi* to extreme osmolarity fluctuations throughout its life cycle. For example, the parasite faces a dramatic drop in osmolarity when it transitions from the hindgut of the triatomine vector (~1,000 mOsm/kg) to the cytosol of the mammalian host (300 mOsm/kg) (7). Modulating the expression levels of TcCARP3 in *T. cruzi* caused significant changes in the ability of parasites to respond to hypoosmotic stress in their extracellular environment. In the absence of TcCARP3, *T. cruzi* epimastigotes displayed an initial swelling and subsequent volume recovery, but these parasites were not able to maintain the cell volume for more than 7 min (420 s) after hypoosmotic stress exposure. This initial volume recovery is compatible with the rapid release of amino acids and other inorganic osmolytes to the extracellular medium, presumably through membrane channels and transporters. This mechanism is responsible for ~50% of cell volume recovery in *T. cruzi* (58). The remaining volume recovery is mediated by the CVC as described above (35, 57). Therefore, the observed *TcCARP3*-KO phenotype is indeed a defect in the CVC-mediated osmoregulatory capacity of the parasites. Conversely, when TcCARP3 is overexpressed, the parasites swell less upon hypoosmotic stress and recover better when compared to control cells. Our results support the hypothesis that abnormal levels of cAMP are generated at the CVC of these mutants. These data support a model of compartmentalized cAMP signals mediating the osmoregulatory capacity of *T. cruzi*. Performing live imaging experiments during hypoosmotic treatment would be necessary to fully understand the dynamics of TcCARP3-TcAC1 interactions at the CVC of *T. cruzi*.

To gain further insight into how TcCARP3 might differentially modulate cAMP synthesis at the flagellar tip and CVC, we analyzed the interactome of TcCARP3 on *T. cruzi* epimastigotes. We previously demonstrated the physical interaction of TcCARP3 and TcAC1 by immunoprecipitation assays and mass spectrometry analysis (17). Here, we confirmed this interaction through co-IP assays and mass spectrometry analysis using TcCARP3 as bait. Mass spectrometry revealed that TcCARP3 not only interacts with TcAC1 but also with seven other ACs from three different groups (TcAC groups I, III, and V). TcAC3 (group III) was previously observed in the ER of epimastigotes, as shown by partial co-localization with the ER marker BiP (17). Considering this new evidence, the localization of TcAC3 should be further investigated to determine if it is indeed localized to the CVC with accumulation in the ER due to overexpression. The *T. brucei* homolog TbCARP3 was also found to interact with multiple ACs in *T. brucei* and to modulate them in different ways depending on the specific identity of the interactor (39). How TcCARP3 interacts with different TcACs and the specific downstream effects arising from these interactions are questions that should be further investigated. In this regard, the predicted TPR-like tetratricopeptide-like helical domain in TcCARP3 (InterPro ID: IPR011990) could be mediating protein-protein interactions (52). Interestingly, this domain is not predicted by InterProScan (59) in the *T. brucei* homolog TbCARP3.

Our cAMP content results are consistent with TcCARP3 being a positive regulator of cAMP levels. However, the metacyclogenesis phenotype of *TcCARP3*-KO parasites is not consistent with the decreased cAMP content of this mutant. Several studies have demonstrated that an increase in cAMP triggers metacyclogenesis *in vitro* (17, 28, 30, 31). The high percentage of MTs observed in *TcCARP3*-KO parasites suggests that, at least in the flagellar tip, the levels of cAMP should be higher in this mutant. Whereas ablation of *TcCARP3* leads to phenotypes compatible with increased cAMP levels in one microdomain (the flagellar tip), our RVD results are consistent with decreased cAMP levels near the CVC of these mutants. Furthermore, the abundance of TcACs detected by mass spectrometry differs between groups (AC3 > AC1 > AC5 isoforms), suggesting that TcCARP3 shows specific affinities for different AC groups. Since trypanosome ACs become catalytically active upon dimerization (60), the monomers to dimers proportion and their composition (homo or heterodimers) could be modulated by TcCARP3-TcAC interactions, determining the TcAC catalytic state and cAMP content in these specific microdomains. We hypothesize that trypanosome AC dimerization state is the indirect consequence of membrane modifications occurring in response to microenvironmental cues, which affect AC-CARP3 interactions at membrane microdomains. Taken together, our results suggest that TcCARP3 is a multi-AC regulator, where AC-CARP3 interaction may determine the catalytic state of different AC dimers. In addition to interacting with several TcACs, our mass spectrometry data indicate that TcCARP3 interacts with the regulatory subunit of a PKA-like protein, a protein that contains two putative cyclic nucleotide binding domains, and therefore could be a cAMP effector. PKArL is the homolog of a divergent PKA regulatory subunit (PKAR3) recently described in *Leishmania donovani* (61). This protein is absent in most trypanosomatids, including *T. brucei*, and is essential for the maintenance of the elongated shape of *Leishmania* promastigotes. The interaction of TcCARP3 with PKArL provides further evidence on the role of TcCARP3 in the cAMP signal transduction pathway in *T. cruzi*. Another putative signaling protein identified in this interactome is Unc119, a protein containing a domain found in GMP-PDE delta subunit and related proteins (IntrePro ID: IPR008015). In *T. brucei*, Unc119 is a myristoylated cargo carrier involved in a conserved lipidated protein intraflagellar transport (LIFT) pathway (62). Unc119 interaction with CARP3 in *T. cruzi* could be necessary for its transport to the flagellar tip, where TcCARP3 interacts with TcAC1 (a transmembrane protein) through its catalytic domain.

The overexpression of TcCARP3 led to a mild increase in total cAMP content compared to control epimastigotes and had no effect on growth and metacyclogenesis. We performed IFAs of *TcCARP3*-OE epimastigotes under normal and hypoosmotic conditions and still observed the dual localization pattern in both. A simple explanation for these results is that TcCARP3 modulates the activity of several TcACs (or at least TcAC1), but these enzymes are expressed at normal levels in TcCARP3-OE parasites, and therefore the overexpression of CARP3 does not significantly affect their activity, as observed when ablating TcCARP3. A possible approach to observe the effect of TcCARP3 overexpression would be the generation of a cell line overexpressing CARP3 and AC1. Nevertheless, TcCARP3-OE MTs were not able to establish an efficient infection in mammalian cells, confirming that cAMP signaling plays a key role in *T. cruzi* infectivity.

The role of cAMP in osmoregulation and metacyclogenesis has been reported by several groups over the last decades (7, 16, 17, 28, 30–35, 57). However, its role in host cell invasion and intracellular replication was first described by our laboratory (17). During the characterization of TcAC1, we showed that increased levels of cAMP lead to a defect in the ability of *T. cruzi* trypomastigotes to invade mammalian host cells and replicate intracellularly as amastigotes. A similar defect was observed in *TcCARP3*-KO parasites showing a lower number of infected host cells at 24 h post-infection and a lower number of intracellular amastigotes after 72 h in mammalian cell cultures. These results further demonstrate that cAMP plays a key role in environmental sensing, with implications for life cycle progression of *T. cruzi* in the mammalian host, as TcCARP3 mutant parasites exhibit decreased levels of cAMP. It would be interesting to evaluate if these mutants are able to establish an acute infection in a murine model. Interestingly, *TcCARP3*-KO parasites showed a defect in colonizing the digestive tract of kissing bugs. Besides their metacyclogenesis phenotype displayed *in vitro*, these parasites were not able to efficiently establish an infection in the insect vector. A possible explanation is that cells undergoing *in vitro* metacyclogenesis in TAU3AAG are subjected to conditions mimicking the vector’s urine composition, but not to the osmolarity the parasite is exposed to in the triatomine bug. These conditions (nutrient deprivation and low pH) (63) trigger differentiation from replicative epimastigotes to infective MTs. However, in the hindgut of the kissing bug, where metacyclogenesis naturally occurs, the microenvironment within the vector reaches an extremely high osmolarity of ~1,000 mOsm/kg (7), while in TAU3AAG medium, the osmolarity is about ~300 mOsm/kg. It is possible that *TcCARP3*-KO parasites do not tolerate the osmotic stress they face in the vector’s gastrointestinal tract, since these parasites showed a reduced osmoregulatory capacity. While the absence of TbCARP3 in the salivarian *T. brucei* was also found to hinder the colonization of tissues in its arthropod vector, the tsetse fly (39, 40), this probably occurs in response to specific microenvironmental cues that are different from those faced by *T. cruzi* in the triatomine vector. Ablation of TbCARP3 did not affect the parasite’s ability to differentiate from procyclic forms to epimastigotes and then to MTs *in vitro*, but caused a defect in SoMo and in the ability of parasites to colonize the tsetse fly salivary glands (39, 40). *T. cruzi,* on the other hand, does not encounter physical barriers in the triatomine vector but does experience intense osmotic stress to which *T. brucei* is never exposed during its life cycle. In this regard, osmotic stress could have been a driving force in the evolutionary retention of the CVC in *T. cruzi*, and for the development of a cAMP signaling microdomain in this subcellular compartment.

Mounting evidence on the role of cAMP in environmental sensing in trypanosomes and in other protozoan parasites has been reported during the last 20 years (7, 16, 17, 20, 28, 34, 35, 40, 44, 64–66). Our data demonstrates that TcCARP3 modulates cAMP levels in *T. cruzi* and is involved in osmoregulation, metacyclogenesis, host cell invasion, intracellular replication, and colonization of the vector’s digestive tract, providing relevant new evidence on the role of cAMP in environmental sensing. We also found that TcCARP3 co-localizes with TcAC1 in all four developmental stages of *T. cruzi* and confirmed direct interaction between these proteins. Together with our proteomic data, these results significantly add to the body of evidence supporting that TcCARP3 is a multi-AC regulator in the flagellar distal domain and the CVC of *T. cruzi*. Future research should be oriented to elucidate the nature of TcCARP3 protein interactions, specifically with ACs and putative cAMP effectors, and to determine how TcCARP3 modulates their activity. Characterizing the signaling components in individual cAMP microdomains is crucial to unveil the essential regulatory mechanisms driving cAMP signaling in trypanosomes.

## MATERIALS AND METHODS

### Chemicals and reagents

Fetal bovine serum (FBS) was purchased from R&D Systems (Minneapolis, MN). G418 was obtained from KSE Scientific (Durham, NC). Puromycin, blasticidin S HCl, Subcloning Efficiency DH5a competent cells, BCA Protein Assay Kit, SuperSignal West Pico Chemiluminescent Substrate, horseradish peroxidase (HRP)-conjugated anti-mouse and anti-rabbit IgG antibodies, mouse anti-HA monoclonal antibody, and Pierce Anti-HA and Anti-c-Myc Magnetic Beads were from Thermo Fisher Scientific (Waltham, MA). Alexa Fluor 488-conjugated donkey anti-mouse and Alexa Fluor 594-conjugated donkey anti-rabbit were from Jackson ImmunoResearch (West Grove, PA). Restriction enzymes and Q5 High-Fidelity DNA Polymerase were obtained from New England BioLabs (Ipswich, MA). ZymoPURE Plasmid Miniprep, ZymoPURE II Plasmid Midiprep, and DNA Clean & Concentrator-5 were from Zymo Research (Irvine, CA). cAMP-Glo Assay Kit, T4 DNA Ligase, and GoTaq G2 Flexi DNA Polymerase were from Promega (Madison, WI). cOmplete Mini EDTA-free Protease Inhibitor Cocktail was from Roche (Basel, Switzerland). 4 mm electroporation cuvettes, Precision Plus Protein Dual Color Standards, and nitrocellulose membranes were from Bio-Rad (Hercules, CA). Mouse anti-c-Myc monoclonal antibody (9E10) was from Santa Cruz Biotechnology (Dallas, TX). Fluoromount-G mounting medium was from Southern Biotech (Birmingham, AL). The pMOTag23M vector (67) was a gift from Dr. Thomas Seebeck (University of Bern, Bern, Switzerland). DNA oligonucleotides were purchased from Integrated DNA Technologies (Coralville, IA). Phenylmethylsulfonyl fluoride (PMSF), N-*p*-tosyl-l-phenylalanine chloromethyl ketone (TPCK), trans-epoxysuccinyl-l-leucylamido-(4-guanidino)butane (E64), protease inhibitor cocktail for use with mammalian cell and tissue extracts (Cat. No. P8340), Benzonase nuclease and all other reagents of analytical grade were from Sigma-Aldrich (St. Louis, MO). Adult *Rhodnius prolixus*, Strain CDC, NR-44077 was provided by the Centers for Disease Control and Prevention for distribution by BEI Resources, NIAID, NIH.

### *In silico* analyses

*TcCARP3* (TriTrypDB gene ID: TcYC6_0045920) sequence and reported tetratricopeptide-like helical domain were retrieved from TriTrypDB.org (46). Proteolipid modification predictions, such as myristoylation prediction, were done using the Research Institute of Molecular Pathology (IMP) NMT—The MYR Predictor (47) and the GPS-Lipid (lipid.biocuckoo.org) tools. Sequence alignment of nucleotides and amino acids was performed using VectorBuilder (vectorbuilder.com). *In silico* restriction enzyme digests, primer designs, and Alpha Fold 3D structure predictions were carried out using Benchling.

### Cell cultures

*T. cruzi* epimastigotes (Y strain) were grown in culture flasks containing liver infusion tryptose (LIT) medium (68) supplemented with 10% heat-inactivated FBS, penicillin (100 IU/mL), and streptomycin (100 µg/mL) at 28°C. Cell density was determined using a Neubauer hemocytometer counting chamber. Control parasites transfected with pTREX-n-3xHA EV and overexpressing cell lines of *TcAC1*-OE and *TcCARP3*-OE were grown in the presence of 250 µg/mL G418. *TcCARP3*-3xc-Myc and *TcCARP3*-3x-Ty1 endogenously tagged cell lines were maintained with 250 µg/mL G418 and 5 µg/mL puromycin. Dually tagged *TcCARP3*-3x-c-Myc/*TcAC1*-3xHA and *TcCARP3*-KO cell lines were grown with 250 µg/mL G418, 5 µg/mL puromycin, and 10 µg/mL blasticidin. *TcCARP3*-AB and *TcCARP3*-8AA were grown in 250 µg/mL G418, 5 µg/mL puromycin, 10 µg/mL blasticidin, and 250 µg/mL Hygromycin. Tissue culture-derived trypomastigotes and amastigotes were collected from the culture medium of infected hFFs cells. hFFs were grown in Dulbecco’s modified Eagle medium (DMEM; Gibco) supplemented with 10% FBS, penicillin (100 IU/mL), and streptomycin (100 µg/mL), and maintained with 5% CO_2_ at 37°C.

### Generation of *TcCARP3* overexpression and dually tagged *TcCARP3*/*TcAC1* parasites

The open reading frame of *TcCARP3* was PCR amplified using *T. cruzi* Y strain gDNA as template (primers 1 and 2; Table S2) and cloned into pTREX-n-3xHA vector (69) by restriction sites XbaI/EcoRV. The construct pTREX-b-*TcAC1*-3xHA for the dually tagged cell line of *TcAC1*-3xHA/*TcCARP3*-3xc-Myc was generated as described in (17). Briefly, amplification of *TcAC1*-3xHA was done by PCR using pTREX-n-*TcAC1*-3xHA plasmid as template and then cloned into pTREX-b by HindIII restriction site using NEBuilder HiFi DNA Assembly Cloning Kit (New England Biolabs). Gene cloning was confirmed by sequencing, and constructs were used to transfect *T. cruzi* epimastigotes. The pTREXn-*TcCARP3*-3xHA construct was used to transfect WT cells to obtain an overexpression cell line *TcCARP3*-OE. Clonal populations were obtained by serial dilutions. The pTREXb-*TcAC1*-3xHA construct was used to transfect endogenously tagged *TcCARP3*-3xc-Myc epimastigotes to obtain a dually tagged cell line. Expression of TcCARP3 and TcAC1 was confirmed by western blot analysis using anti-c-Myc and anti-HA antibodies, respectively.

### Ablation of *TcCARP3*

We performed a CRISPR/Cas9-mediated knock out of *TcCARP3* using a standard strategy developed in our laboratory (70). Briefly, *T. cruzi* Y strain epimastigotes constitutively expressing T7 RNA polymerase and Cas9 were transfected with a sgRNA template obtained by PCR (primers 3 and 17; Table S2) and two donor DNA cassettes amplified from pGEM-BSD-TGA and pGEM-PAC-TGA (primers 4 and 5; Table S2), respectively. The donor DNA was provided to induce homology-directed repair (HDR) and contained a blasticidin or a puromycin resistance marker, respectively, flanked by 40 and 37 nt homologous regions corresponding to the 5′ and 3′ end of the *TcCARP3* UTRs. Selection of the protospacer was performed using EuPaGDT (eukaryotic pathogen CRISPR guide RNA/DNA design tool; http://grna.ctegd.uga.edu) (58). We chose a specific sgRNA sequence targeting a site within the ORF of the *TcCARP3* gene. Selection of transfectants was done with puromycin and blasticidin to ensure both alleles were replaced by resistance markers. Gene knockout was verified by PCR from gDNA using a specific set of primers (primers 6 and 7; Table S2). After *TcCARP3* knockout was confirmed, a clonal population was obtained by serial dilutions.

### Generation of addback cell lines

We obtained the addback cell line by amplifying the ORF of *TcCARP3* using pTREXn-*TcCARP3*-3xHA as a template and subcloning it into pTREXh-2xTy1 through restriction sites XbaI/EcoRV (primers 1 and 8; Table S2). This construct was then used to transfect *TcCARP3*-KO parasites to obtain the *TcCARP3* addback (*TcCARP3*-AB). We obtained another construct with a truncated version of TcCARP3 where the first eight amino acids had been deleted to get rid of the first three glycine residues that contained a predicted myristoylation site. To achieve this, the sequence downstream of the eighth codon (24 nt) of *TcCARP3* was amplified using pTREXn-*TcCARP3*-3xHA as a template and subcloned into pTREXh-2xTy1 through restriction sites XbaI/EcoRV (primers 8 and 9; Table S2). The construct was then transfected into *TcCARP3*-KO parasites to obtain the *TcCARP3*-8AA cell line. Both constructs were verified by restriction digestion and Sanger sequencing before transfection. Successfully transfected parasites were confirmed by western blot and IFA after selection with hygromycin.

### Endogenous tagging of *TcCARP3*

We performed a CRISPR/Cas9-mediated endogenous C-terminal tagging of *TcCARP3*. Briefly, *T. cruzi* Y strain epimastigotes constitutively expressing T7 RNA polymerase and Cas9 were transfected with a sgRNA template obtained by PCR (primers 10 and 17; Table S2) and a donor DNA cassette, amplified from pMOTag23T vector (primers 11 and 12; Table S2). The pMOTag23T vector was made by amplifying two copies of the Ty1 tag from the pTREXh-2xTy1 vector (primers 13 and 14; Table S2) and cloned into pMOTag2T already containing a puromycin resistance marker and one copy of the Ty1 tag (67) by XhoI site using NEBuilder HiFi DNA Assembly Cloning Kit (New England Biolabs). The donor DNA provided to induce homology-directed repair contained a 3xTy1 tag, a puromycin resistance marker, and 65 and 60 nt homologous regions at the 5 ′and 3′ ends of the cassette, respectively. Selection of the protospacer was performed using EuPaGDT. We chose a specific sgRNA sequence targeting the 3′ end of the *TcCARP3* gene. Selection of transfectants was done with puromycin. Endogenous gene tagging was verified by PCR from gDNA using a specific set of primers (primers 15 and 16; Table S2) and by western blot analysis.

### Transfection of *T. cruzi* epimastigotes

*T. cruzi* Y strain epimastigotes were transfected via electroporation as previously described (69). Briefly, 4 × 10^7^ cells in early exponential phase were washed with sterile 1× PBS pH 7.4 at RT and resuspended in ice-cold CytoMix (120 mM KCl, 0.15 mM CaCl_2_, 10 mM K_2_HPO_4_, 25 mM HEPES, 2 mM EDTA, and 5 mM MgCl_2_, pH 7.6) to a final density of 1 × 10^8^ cells/mL. Then, 400 µL of cell suspension was transferred to a cold 4 mm electroporation cuvette that was on ice containing 25 µg of each DNA fragment (purified plasmid or PCR product) in a maximum DNA volume of 40 μL. Three electric pulses (1500 V, 25 µF) were sent to the cells in cuvettes, using a Gene Pulser Xcell Electroporation System (Bio-Rad). Transfected epimastigotes were cultured in LIT medium supplemented with 20% heat-inactivated FBS and the corresponding antibiotics for selection of successfully transfected parasites expressing antibiotic resistance, until healthy cell lines were obtained (2–3 weeks). Clonal populations of transfectant parasites were obtained by serial dilutions in LIT medium and a final dilution in conditioned media (20% heat inactivated FBS, 40% filtered supernatant from WT cells in exponential phase, 40% LIT media, penicillin [100 IU/mL], streptomycin [100 µg/mL], and appropriate antibiotics) to a final density of 2.5 cells/mL and plated 200 µL per well in 96-well plates.

### Western blot analyses

Western blots were performed as previously described (71). Briefly, parasites in the exponential phase of growth were washed in 1× PBS pH 7.4 and resuspended in radio-immunoprecipitation assay (RIPA) buffer (150 mM NaCl, 20 mM Tris-HCl, pH 7.5, 1 mM EDTA, 1% SDS, and 0.1% Triton X-100) plus a mammalian cell protease inhibitor cocktail (diluted 1:250), 1 mM phenylmethylsulfonyl fluoride, 2.5 mM tosyl phenylalanyl chloromethyl ketone, 100 M *N*-(*trans*-epoxysuccinyl)-l-leucine 4-guanidinobutylamide (E64), and benzonase nuclease (25 U/mL culture). After lysis, the cells were then incubated for 30 min on ice, and protein concentration was determined by BCA Protein Assay. Thirty micrograms of protein from each cell lysate were mixed with 4× Laemmli sample buffer (Bio-Rad) supplemented with 10% β-mercaptoethanol, before loading into 10%, 8%, or 6% SDS–polyacrylamide gels. Electrophoresed proteins were then transferred onto nitrocellulose membranes with a Trans-Blot Turbo Transfer System (Bio-Rad). After transfer, the membranes were stained with Ponceau red, and an image was acquired for loading control using a ChemiDoc Imaging System (Bio-Rad). Membranes were then destained using PBS-T (PBS containing 0.1% Tween 20) and blocked with 5% nonfat dry milk in PBS-T overnight at 4°C. Then, the nitrocellulose membranes were incubated for 1 h at room temperature, with the primary antibody: HA tag monoclonal antibody 2–2.2.14 (1:2,000), anti-c-Myc monoclonal antibody 9E10 (1:1,000), or Ty1 tag monoclonal antibody BB2 (1:2,000). After three washes with PBS-T, blots were incubated with the secondary HRP-conjugated antibody (goat anti-mouse IgG or goat anti-rabbit IgG, diluted 1:10,000). Membranes were washed three times with PBS-T and incubated with Pierce ECL Western Blotting Substrate (Thermo Fisher Scientific) in the dark for 5 min. Finally, images were acquired with a ChemiDoc Imaging System (Bio-Rad).

### IFAs

*T. cruzi* parasites (epimastigotes, trypomastigotes, or amastigotes) were washed with 1× PBS pH 7.4 and fixed with 4% paraformaldehyde (PFA) in 1× PBS pH 7.4 for 1 h at RT. IFAs involving TcAC1 and TcCARP3 mutants were performed under hypoosmotic conditions, by adding an equal volume of deionized water to the parasites in 1× PBS pH 7.4 and fixing them after exactly 2 min. Thereafter, cells were allowed to adhere to 1 mg/mL poly-l-lysine-coated coverslips and then permeabilized for 5 min with 0.1% Triton X-100. The coverslips were then washed three times with 1× PBS pH 7.4. The cells were then blocked with trypanosome blocking solution (3% bovine serum albumin [BSA], 1% fish gelatin, 5% normal goat serum, and 50 mM NH_4_Cl, in PBS pH 7.4), overnight at 4°C. Next, the cells were incubated with primary antibodies: rabbit anti-HA polyclonal antibody SG77 (1:200) and/or anti-c-Myc monoclonal antibody 9E10 (1:100), diluted in 1% BSA in 1× PBS pH 8.0 for 1 h at RT. The cells were washed three times with 1% BSA in 1× PBS pH 8.0 and then incubated for 1 h at RT with secondary antibodies: Alexa Fluor 488-conjugated donkey anti-mouse (1:400) and/or Alexa Fluor 594-conjugated donkey anti-rabbit (1:400). The incubation was performed keeping the cells protected from light to avoid photobleaching. Then, the cells were washed three times with 1% BSA in 1× PBS pH 8.0 and mounted on slides using Fluoromount-G mounting medium containing 5 µg/mL 4,6-diamidino-2-phenylindole (DAPI) to stain genetic material. Differential interference contrast (DIC) and fluorescence optical images were captured using a Nikon Ni-E epifluorescence microscope on 100× oil immersion lens using NIS-Elements software for acquisition and subsequent processing of the images.

### Determination of cAMP content

Intracellular levels of cAMP in *T. cruzi* epimastigotes were determined using the luminescent assay cAMP-Glo (Promega) following the manufacturer’s protocol. Briefly, *T. cruzi* epimastigotes in exponential phase of growth were washed two times with 1× PBS pH 7.4 and resuspended in induction buffer (500 mM 3-Isobutyl-1-methylxanthine and 100 mM Ro 20-1724 in PBS, pH 7.4) to a final density of 1 × 10^9^ cells/mL. Next, 10 mL of cell suspension was transferred into a white 96-well plate in triplicates (1 × 10^7^ cells/well). A portion of the total lysate was used to quantify protein concentration using the BCA Assay. Cells in wells were lysed by adding 10 mL of cAMP-Glo lysis buffer and incubating them at RT for 15 min. Next, 20 µL of cAMP detection solution was added to each well. Cells in the plate were agitated for 1 min in an orbital shaker and incubated for 20 min at RT. Finally, 40 µL of Kinase-Glo Reagent was simultaneously added to the wells. After shaking for 1 min, the plate was incubated for 10 min at RT. Luminescence was measured using a BioTek Synergy H1 Plate Reader (Agilent Technologies). Results were expressed as mean values of cAMP content relative to control cells from three independent experiments and normalized by protein concentration.

### RVD assays

RVD after hypoosmotic stress was monitored as described previously (17, 42). Briefly, *T. cruzi* epimastigotes in exponential phase of growth were centrifuged at 1,000 × *g* for 7 min, washed two times in 1× PBS pH 7.4, and resuspended in isosmotic buffer (64 mM NaCl, 4 mM KCl, 1.8 mM CaCl_2_, 0.53 mM MgCl_2_, 5.5 mM glucose, 150 mM d-mannitol, 5 mM HEPES-Na, pH 7.4, and 282 mOsmol/L) at a cell density of 1 × 10^8^ cells/mL. Next, 100 µL was aliquoted in a 96-well plate in triplicates, and the absorbance at 550 nm was measured every 10 s for 3 min using a BioTek Synergy H1 Plate Reader (Agilent Technologies). Then, 200 µL of hypoosmotic buffer (64 mM NaCl, 4 mM KCl, 1.8 mM CaCl_2_, 0.53 mM MgCl_2_, 5.5 mM glucose, and 5 mM HEPES-Na, pH 7.4) was added simultaneously for a final osmolarity of 115 mOsmol/L, and the absorbance at 550 nm was measured after hypoosmotic stress for an additional 12 min. Readings were normalized using the mean value of the initial 3 min in isosmotic buffer. Normalized 550 nm absorbance readings were then converted into a percent volume change using the equation: (|*V*_f_ – *V*_o_|/*V*_o_) × 100, where *V*_f_ is the absorbance value at the time point after hypoosmotic stress and Vo is the absorbance mean value obtained under isosmotic conditions. The osmoregulatory capacity of *T. cruzi* cell lines was quantified using two different parameters: the maximum change of cell volume upon induction of hypoosmotic stress (AUC between 200 and 300 s in the absorbance chart) and the final volume recovery (AUC between 700 and 800 s).

### *In vitro* metacyclogenesis

MTs were obtained following the protocol described in reference 63 with some modifications. Briefly, *T. cruzi* epimastigotes were cultured for 4 days in LIT medium supplemented with 10% heat-inactivated FBS. The parasites were then washed two times in 2 mL of triatome artificial urine (TAU) (190 mM NaCl, 17 mM KCl, 2 mM MgCl_2_, 2 mM CaCl_2_, 0.035% sodium bicarbonate, and 8 mM phosphate, pH 6.9) and resuspended in 0.2 mL of TAU medium. Parasites were then incubated for 2 h at 28°C. After incubation, parasites were added to flasks and incubated horizontally for 96 h in 20 mL TAU 3AAG medium (TAU medium supplemented with 10 mM l-proline, 50 mM sodium l-glutamate, 2 mM sodium l-aspartate, and 10 mM glucose) in T25 flasks. For quantification of metacyclogenesis, the supernatant containing a mixture of epimastigotes, MTs, and intermediate forms was centrifuged at 1,300 × *g* for 15 min and fixed for 1 h at RT in 4% PFA in PBS, attached to poly-l-Lysine-coated coverslips and washed three times with 1× PBS pH 7.4. Then, parasites were mounted onto glass slides with Fluoromount-G containing 15 µg/mL DAPI, for DNA staining. Twenty fields/slide were analyzed on a Nikon epifluorescence microscope with a 100× objective under oil immersion in three independent experiments. MTs were distinguished from epimastigotes by the kinetoplast location in the cell body. The kinetoplast is more posterior in MTs, while in epimastigotes, it is located between the nucleus and the flagellum. After confirmation of the kinetoplast location, DIC was used to confirm the slender morphology consistent with a MT.

### Adhesion assays

During *in vitro* metacyclogenesis, parasites adhere to the plastic within the first 6 h of horizontal incubation in TAU 3AAG medium. Thereafter, fully differentiated MTs spontaneously detach and are released into the TAU 3AAG during the following 96 h (28). To assess the ability of *T. cruzi* epimastigotes to adhere to the plastic in a sterile 12-well plates during the incubation in TAU 3AAG medium, parasite density in the medium was determined at 2, 4, 6, 24, 48, 72, and 96 h using a Neubauer hemocytometer counting chamber. The number of parasites that were adhered was determined by subtracting the total number of non-adhered cells using the density calculated and total volume added from the initial number of cells added to the well.

### Host cell invasion and intracellular replication

*T. cruzi* invasion and intracellular replication assays were performed using hFFs. First, 5 × 10^4^ hFFs in 1 mL of DMEM supplemented with 10% FBS were added to 12-well plates containing a sterile coverslip and allowed to attach overnight at 37°C with 5% CO_2_. The next day, a swimming protocol was performed on tissue culture-derived trypomastigotes by centrifuging at 1,700 × *g* for 15 min and incubating upright in a 50 mL conical tube for 4 h at 37°C with 5% CO_2_. This allowed the competent trypomastigotes to swim out of the pellet into the supernatant. Next, the supernatant was spun down, and the density of parasites was determined using a Neubauer chamber and resuspended to a concentration of 5 × 10^6^ parasites/mL. The hFFs in the 12-well plate were washed with DHANKS (Hank’s Balanced Salt Solution, Cytiva Marlborough, MA), and 1 mL of the parasite suspension (5 × 10^6^ parasites) was added for a multiplicity of infection (MOI) of 100 (100 trypomastigotes/cell). The infection was stopped after 4 h by washing the coverslips in the wells five times with DHANKS. Then, 1 mL of DMEM with 2% FBS was added to slow down the proliferation of hFFs. Coverslips were removed from the plate after 24 h for the invasion assay and after 72 h for the replication assay and placed into a 12-well plate containing 4% paraformaldehyde in 1× PBS pH 7.4 for 1 h. The coverslips were then washed with 1× PBS pH 7.4 and mounted onto glass slides containing 15 µg/mL DAPI in Fluoromount G for DNA staining of parasites and mammalian cells. To quantify invasion, 20 fields/slide were visualized on a Nikon Ni-E epifluorescence microscope, and the number of infected and non-infected cells was counted. To quantify the replication of amastigotes, 60 infected host cells were visualized per assay on a Nikon Ni-E epifluorescence microscope, and the number of amastigotes per infected cell was counted.

### Co-immunoprecipitation of TcAC1-3xHA/TcCARP3-3xc-Myc

*T. cruzi* epimastigotes (2 × 10^8^ cells) in exponential phase of growth were centrifuged at 1,000 × *g* for 15 min and washed two times with 5 mL of buffer A with glucose (BAG, 116 mM NaCl, 5.4 mM KCl, 0.8 mM MgSO_4_, 50 mM HEPES, and 5.5 mM glucose, pH 7.3) at room temperature. The parasites were then resuspended in 1 mL of ice-cold lysis buffer (0.4% NP-40, 1 mM EDTA, 150 mM KCl, cOmplete Mini EDTA-free Protease Inhibitor Cocktail, and 50 mM Tris-HCl, pH 7.5) and mixed at 4°C for 30 min with agitation on a rocking shaker. After lysis, the protein concentration of each sample was determined using the BCA Assay. Cell lysate was centrifuged at 4°C at 15,000 × *g* for 20 min and the supernatant was incubated with 50 µL of Pierce Anti-HA magnetic beads to trap TcAC1-3xHA as the bait protein, or Pierce Anti-c-Myc magnetic beads to trap TcCARP3-3xc-Myc as the bait protein. The beads had been previously washed with lysis buffer using a magnetic rack, and the amount of protein loaded into the tubes with the magnetic beads was standardized based on BCA protein quantification. A portion of the pre-cleared lysate (PCL) was saved for subsequent western blot analysis. The soluble fraction of the supernatant was then incubated with magnetic beads for 1 h at RT with gentle agitation. After incubation, the flow-through was removed and saved, and the magnetic beads were then washed three times with wash buffer (0.1% NP-40, 1 mM EDTA, 150 mM KCl, cOmplete Mini EDTA-free Protease Inhibitor Cocktail, and 50 mM Tris-HCl, pH 7.5) using a magnetic rack. The third wash was saved for subsequent western blot analysis. A final wash was performed using pure deionized water. Proteins were then eluted with 100 µL of elution buffer (0.1 M glycine, pH 2.0), by applying gentle agitation for 10 min at RT. Eluates were then neutralized with 15 µL neutralization buffer (1 M Tris, pH 9.5) and analyzed by western blot with anti-HA antibodies for the anti-c-Myc IP to detect TcAC1-3x-HA as prey, or with anti-c-Myc antibodies for the anti-HA IP to detect TcCARP3-3x-c-Myc as prey.

### Analysis of TcCARP3 interactome

We performed immunoprecipitation of TcCARP3-3xHA overexpression and pTREXn-3xHA EV cell lines using Pierce anti-HA magnetic beads. The same general procedure was followed as described above in the “Co-immunoprecipitation of TcAC1-3xHA/TcCARP3-3xc-Myc” section. Eluted fractions from TcCARP3-3xHA overexpressing parasites and EV control cells were sent to the Mass Spectrometry Core Laboratory at The University of Texas Health Science Center (San Antonio, TX) for analysis. Aliquots of the eluates (100 µL) were mixed with 100 µL 10% SDS in 50 mM triethylammonium bicarbonate (TEAB), reduced with tris(2-carboxyethyl)phosphine hydrochloride , alkylated in the dark with iodoacetamide, and applied to S-Traps micro (ProtiFi) for tryptic digestion (sequencing grade; Promega) for 2 h in 50 mM TEAB. Peptides were eluted from each S-Trap with 0.2% formic acid in 50% aqueous acetonitrile. Digests were analyzed by capillary HPLC-electrospray ionization tandem mass spectrometry on a Thermo Scientific Orbitrap Fusion Lumos mass spectrometer. On-line HPLC separation was accomplished with an RSLC NANO HPLC system (Thermo Scientific/Dionex) interfaced with a Nanospray Flex ion source (Thermo Scientific) fitted with a PepSep column (Bruker; ReproSil C18, 15 cm × 150 µm, 1.9 µm beads). Precursor ions were acquired in the Orbitrap in centroid mode at 120,000 resolution (*m/z* 200); data-dependent higher-energy collisional dissociation spectra were acquired at the same time in the linear trap using the “rapid" speed option (30% normalized collision energy). Mascot (v2.8.3; Matrix Science, London UK) was used to search the spectra against a combination of the following databases: TcruziYC6_TriTrypDB-67 20240206, a “local” database that includes the sequences of recombinant and target proteins, antibodies used for pull-down experiments, and common contaminants. Cysteine carbamidomethylation was set as a fixed modification, and methionine oxidation and deamidation of glutamine and asparagine were considered as variable modifications; trypsin was specified as the proteolytic enzyme, with two missed cleavages allowed. The Mascot search results were imported into Scaffold (version 5.3.3, Proteome Software Inc., Portland, OR). A minimum of two identified peptides was required. The settings used resulted in a protein-level FDR of 0.3%. The top interactors of TcCARP3 were identified based on the enrichment of a protein in the TcCARP3-OE cell line eluate and absence in the EV eluate. Criteria for top interactors of TcCARP3 were infinite fold change (interacting protein present in all three of the TcCARP3-OE replicates and absent in the EV replicates analyzed), a total spectra count ≥1 in each of the three *TcCARP3*-OE replicates, and a *P* value < 0.05 after a *t*-test based on total spectra count, with Benjamini-Hochberg multiple test correction.

### Infection of kissing bugs with *T. cruzi* parasites

Kissing bugs (*R. prolixus*) were obtained from the colonies established at the Centers for Disease Control and Prevention (BEI Resources, NR-44077) (72). These kissing bugs were fed artificially bi-weekly with defibrinated rabbit blood (Hemostat) with a parafilm membrane feeding system (Hemotek). The colony condition was held at 24.0°C, 50 ± 10% relative humidity, and 6:00 am/6:00 pm light/dark photoperiods. Third instar *R. prolixus* were collected for infection with parasites. *T. cruzi* epimastigotes in the exponential phase of growth were washed in 5 mL of 1× PBS pH 7.4 and mixed with defibrinated rabbit blood (complement inactivated at 56 ± 0.5°C before the addition of parasites) and offered to triatomines at 37°C through an artificial feeder at a concentration of 1 × 10^8^ parasites/mL (73–75). The kissing bugs were held at 24 ± 0.5°C, 50 ± 10% relative humidity, and 6:00 am/6:00 pm light/dark photoperiods to allow for parasite growth and differentiation. After four weeks, the hindguts were dissected out of the triatomine bugs, emulsified in 100 µL of 1× PBS pH 7.4, and examined under the microscope for the presence of parasites in the hindgut to establish the percentage of infected insects. Three groups of 9–15 infected kissing bugs were dissected per *T. cruzi* cell line, which vary due to the number of kissing bugs feeding with offered blood with trypanosomes.

### Statistical analyses

Values are expressed as means ± standard deviation (SD). Statistically significant differences between treatments were compared using unpaired Student’s *t*-test, Kruskal-Wallis test, and one-way and two-way ANOVA tests with multiple comparisons, as mentioned in the legends of the figures. Differences were considered statistically significant for *P* < 0.05, and *n* refers to the number of independent experiments performed. All statistical analyses were performed using GraphPad Prism 9 (GraphPad Software, San Diego, CA).

## SUPPLEMENTAL MATERIAL

Supplemental Figures mbio.00994-25-s0001.pdf Figures S1 to S4.

Table S1 mbio.00994-25-s0002.xlsx TcCARP3 specific protein interactors identified by mass spectrometry analysis.

Table S2 mbio.00994-25-s0003.pdf List of oligonucleotides used in this study.

ASM does not own the copyrights to Supplemental Material that may be linked to, or accessed through, an article. The authors have granted ASM a non-exclusive, world-wide license to publish the Supplemental Material files. Please contact the corresponding author directly for reuse.

## References

[1] Forsyth C, Agudelo Higuita NI, Hamer SA, Ibarra-Cerdeña CN, Valdez-Tah A, Stigler Granados P, Hamer GL, Vingiello M, Beatty NL. 2024. Climate change and Trypanosoma cruzi transmission in North and central America. Lancet Microbe 5:100946. doi:10.1016/j.lanmic.2024.07.009 39284331

[2] Agudelo Higuita NI, Beatty NL, Forsyth C, Henao-Martínez AF, Manne-Goehler J, US Chagas Research Consortium. 2024. Chagas disease in the United States: a call for increased investment and collaborative research. Lancet Reg Health Am 34:100768. doi:10.1016/j.lana.2024.100768 38798947 PMC11127192

[3] Nunes MCP, Beaton A, Acquatella H, Bern C, Bolger AF, Echeverría LE, Dutra WO, Gascon J, Morillo CA, Oliveira-Filho J, Ribeiro ALP, Marin-Neto JA, American Heart Association Rheumatic Fever, Endocarditis and Kawasaki Disease Committee of the Council on Cardiovascular Disease in the Young; Council on Cardiovascular and Stroke Nursing; and Stroke Council. 2018. Chagas cardiomyopathy: an update of current clinical knowledge and management: a scientific statement from the American heart association. Circulation 138:e169–e209. doi:10.1161/CIR.0000000000000599 30354432

[4] Bern C, Messenger LA, Whitman JD, Maguire JH. 2019. Chagas disease in the United States: a public health approach. Clin Microbiol Rev 33:e00023-19. doi:10.1128/CMR.00023-19 31776135 PMC6927308

[5] Povelones ML, Holmes NA, Povelones M. 2023. A sticky situation: when trypanosomatids attach to insect tissues. PLoS Pathog 19:e1011854. doi:10.1371/journal.ppat.1011854 38128049 PMC10734937

[6] Melo R de FP, Guarneri AA, Silber AM. 2020. The influence of environmental cues on the development of Trypanosoma cruzi in triatominae vector. Front Cell Infect Microbiol 10:27. doi:10.3389/fcimb.2020.00027 32154185 PMC7046586

[7] Lander N, Chiurillo MA, Docampo R. 2021. Signaling pathways involved in environmental sensing in Trypanosoma cruzi. Mol Microbiol 115:819–828. doi:10.1111/mmi.14621 33034088 PMC8032824

[8] Schoijet AC, Sternlieb T, Alonso GD. 2019. Signal transduction pathways as therapeutic target for chagas disease. CMC 26:6572–6589. doi:10.2174/0929867326666190620093029 31218950

[9] Wang L, Wu C, Peng W, Zhou Z, Zeng J, Li X, Yang Y, Yu S, Zou Y, Huang M, et al.. 2022. A high-performance genetically encoded fluorescent indicator for in vivo cAMP imaging. Nat Commun 13:5363. doi:10.1038/s41467-022-32994-7 36097007 PMC9468011

[10] Musheshe N, Schmidt M, Zaccolo M. 2018. cAMP: from long-range second messenger to nanodomain signalling. Trends Pharmacol Sci 39:209–222. doi:10.1016/j.tips.2017.11.006 29289379

[11] Khannpnavar B, Mehta V, Qi C, Korkhov V. 2020. Structure and function of adenylyl cyclases, key enzymes in cellular signaling. Curr Opin Struct Biol 63:34–41. doi:10.1016/j.sbi.2020.03.003 32334344

[12] Adderley SP, Sprague RS, Stephenson AH, Hanson MS. 2010. Regulation of cAMP by phosphodiesterases in erythrocytes. Pharmacol Rep 62:475–482. doi:10.1016/s1734-1140(10)70303-0 20631411 PMC2922877

[13] D’Angelo MA, Montagna AE, Sanguineti S, Torres HN, Flawiá MM. 2002. A novel calcium-stimulated adenylyl cyclase from Trypanosoma cruzi, which interacts with the structural flagellar protein paraflagellar rod. J Biol Chem 277:35025–35034. doi:10.1074/jbc.M204696200 12121994

[14] D’Angelo MA, Sanguineti S, Reece JM, Birnbaumer L, Torres HN, Flawiá MM. 2004. Identification, characterization and subcellular localization of TcPDE1, a novel cAMP-specific phosphodiesterase from Trypanosoma cruzi. Biochem J 378:63–72. doi:10.1042/BJ20031147 14556647 PMC1223918

[15] Díaz-Benjumea R, Laxman S, Hinds TR, Beavo JA, Rascón A. 2006. Characterization of a novel cAMP-binding, cAMP-specific cyclic nucleotide phosphodiesterase (TcrPDEB1) from Trypanosoma cruzi*.* Biochem J 399:305–314. doi:10.1042/BJ20060757 16776650 PMC1609912

[16] Schoijet AC, Miranda K, Medeiros LCS, de Souza W, Flawiá MM, Torres HN, Pignataro OP, Docampo R, Alonso GD. 2011. Defining the role of a FYVE domain in the localization and activity of a cAMP phosphodiesterase implicated in osmoregulation in Trypanosoma cruzi. Mol Microbiol 79:50–62. doi:10.1111/j.1365-2958.2010.07429.x 21166893 PMC3056490

[17] Chiurillo MA, Carlson J, Bertolini MS, Raja A, Lander N. 2023. Dual localization of receptor-type adenylate cyclases and cAMP response protein 3 unveils the presence of two putative signaling microdomains in Trypanosoma cruzi. MBio 14:e0106423. doi:10.1128/mbio.01064-23 37477489 PMC10470820

[18] Lopez MA, Saada EA, Hill KL. 2015. Insect stage-specific adenylate cyclases regulate social motility in African trypanosomes. Eukaryot Cell 14:104–112. doi:10.1128/EC.00217-14 25416239 PMC4279026

[19] Saada EA, Kabututu ZP, Lopez M, Shimogawa MM, Langousis G, Oberholzer M, Riestra A, Jonsson ZO, Wohlschlegel JA, Hill KL. 2014. Insect stage-specific receptor adenylate cyclases are localized to distinct subdomains of the Trypanosoma brucei flagellar membrane. Eukaryot Cell 13:1064–1076. doi:10.1128/EC.00019-14 24879126 PMC4135804

[20] Shaw S, DeMarco SF, Rehmann R, Wenzler T, Florini F, Roditi I, Hill KL. 2019. Flagellar cAMP signaling controls trypanosome progression through host tissues. Nat Commun 10:803. doi:10.1038/s41467-019-08696-y 30778051 PMC6379439

[21] Bachmaier S, Volpato Santos Y, Kramer S, Githure GB, Klöckner T, Pepperl J, Baums C, Schenk R, Schwede F, Genieser H-G, Dupuy J-W, Forné I, Imhof A, Basquin J, Lorentzen E, Boshart M. 2019. Nucleoside analogue activators of cyclic AMP-independent protein kinase A of Trypanosoma. Nat Commun 10:1421. doi:10.1038/s41467-019-09338-z 30926779 PMC6440977

[22] Ober VT, Githure GB, Volpato Santos Y, Becker S, Moya Munoz G, Basquin J, Schwede F, Lorentzen E, Boshart M. 2024. Purine nucleosides replace cAMP in allosteric regulation of PKA in trypanosomatid pathogens. Elife 12:RP91040. doi:10.7554/eLife.91040 38517938 PMC10959531

[23] Seebeck T, Schaub R, Johner A. 2004. cAMP signalling in the kinetoplastid protozoa. Curr Mol Med 4:585–599. doi:10.2174/1566524043360113 15357210

[24] Laxman S, Beavo JA. 2007. Cyclic nucleotide signaling mechanisms in trypanosomes: possible targets for therapeutic agents. Mol Interv 7:203–215. doi:10.1124/mi.7.4.7 17827441

[25] Salmon D. 2018. Adenylate cyclases of Trypanosoma brucei, environmental sensors and controllers of host innate immune response. Pathogens 7:48. doi:10.3390/pathogens7020048 29693583 PMC6027212

[26] Tagoe DNA, Kalejaiye TD, de Koning HP. 2015. The ever unfolding story of cAMP signaling in trypanosomatids: vive la difference! Front Pharmacol 6:185. doi:10.3389/fphar.2015.00185 26441645 PMC4561360

[27] Lander N. 2024. mSphere of influence: compartmentalized cAMP signals in American trypanosomes. mSphere 9:e0063523. doi:10.1128/msphere.00635-23 38315033 PMC10900897

[28] Hamedi A, Botelho L, Britto C, Fragoso SP, Umaki ACS, Goldenberg S, Bottu G, Salmon D. 2015. In vitro metacyclogenesis of Trypanosoma cruzi induced by starvation correlates with a transient adenylyl cyclase stimulation as well as with a constitutive upregulation of adenylyl cyclase expression. Mol Biochem Parasitol 200:9–18. doi:10.1016/j.molbiopara.2015.04.002 25912925

[29] Garcia ES, Gonzalez MS, de Azambuja P, Baralle FE, Fraidenraich D, Torres HN, Flawiá MM. 1995. Induction of Trypanosoma cruzi metacyclogenesis in the gut of the hematophagous insect vector, Rhodnius prolixus, by hemoglobin and peptides carrying alpha D-globin sequences. Exp Parasitol 81:255–261. doi:10.1006/expr.1995.1116 7498422

[30] Rangel-Aldao R, Triana F, Comach G, Abate T, Fernández V, McMahon-Pratt D. 1988. Intracellular signaling transduction in the differentiation of Trypanosoma cruzi: role of cAMP. Arch Biol Med Exp 21:403–408. 2855697

[31] Rangel-Aldao R, Triana F, Fernández V, Comach G, Abate T, Montoreano R. 1988. Cyclic AMP as an inducer of the cell differentiation of Trypanosoma cruzi*.* Biochem Int 17:337–344. 2847739

[32] Gonzales-Perdomo M, Romero P, Goldenberg S. 1988. Cyclic AMP and adenylate cyclase activators stimulate Trypanosoma cruzi differentiation. Exp Parasitol 66:205–212. doi:10.1016/0014-4894(88)90092-6 2840306

[33] Docampo R, Jimenez V, Lander N, Li ZH, Niyogi S. 2013. New insights into roles of acidocalcisomes and contractile vacuole complex in osmoregulation in protists. Int Rev Cell Mol Biol 305:69–113. doi:10.1016/B978-0-12-407695-2.00002-0 23890380 PMC3818246

[34] King-Keller S, Li M, Smith A, Zheng S, Kaur G, Yang X, Wang B, Docampo R. 2010. Chemical validation of phosphodiesterase C as a chemotherapeutic target in Trypanosoma cruzi, the etiological agent of chagas’ disease. Antimicrob Agents Chemother 54:3738–3745. doi:10.1128/AAC.00313-10 20625148 PMC2934955

[35] Rohloff P, Montalvetti A, Docampo R. 2004. Acidocalcisomes and the contractile vacuole complex are involved in osmoregulation in Trypanosoma cruzi. Journal of Biological Chemistry 279:52270–52281. doi:10.1074/jbc.M410372200 15466463

[36] Augusto I, Girard-Dias W, Schoijet A, Alonso GD, Portugal RV, de Souza W, Jimenez V, Miranda K. 2024. Quantitative assessment of the nanoanatomy of the contractile vacuole complex in Trypanosoma cruzi. Life Sci Alliance 7:e202402826. doi:10.26508/lsa.202402826 39074903 PMC11287019

[37] Gould MK, Bachmaier S, Ali JAM, Alsford S, Tagoe DNA, Munday JC, Schnaufer AC, Horn D, Boshart M, de Koning HP. 2013. Cyclic AMP effectors in African trypanosomes revealed by genome-scale RNA interference library screening for resistance to the phosphodiesterase inhibitor CpdA. Antimicrob Agents Chemother 57:4882–4893. doi:10.1128/AAC.00508-13 23877697 PMC3811416

[38] Bachmaier S, Gould MK, Polatoglou E, Omelianczyk R, Brennand AE, Aloraini MA, Munday JC, Horn D, Boshart M, de Koning HP. 2023. Novel kinetoplastid-specific cAMP binding proteins identified by RNAi screening for cAMP resistance in Trypanosoma brucei. Front Cell Infect Microbiol 13:1204707. doi:10.3389/fcimb.2023.1204707 37475965 PMC10354285

[39] Bachmaier S, Giacomelli G, Calvo-Alvarez E, Vieira LR, Van Den Abbeele J, Aristodemou A, Lorentzen E, Gould MK, Brennand A, Dupuy J-W, Forné I, Imhof A, Bramkamp M, Salmon D, Rotureau B, Boshart M. 2022. A multi-adenylate cyclase regulator at the flagellar tip controls African trypanosome transmission. Nat Commun 13:5445. doi:10.1038/s41467-022-33108-z 36114198 PMC9481589

[40] Shaw S, Knüsel S, Abbühl D, Naguleswaran A, Etzensperger R, Benninger M, Roditi I. 2022. Cyclic AMP signalling and glucose metabolism mediate pH taxis by African trypanosomes. Nat Commun 13:603. doi:10.1038/s41467-022-28293-w 35105902 PMC8807625

[41] Schaub GA, Kleffmann T, Kollien AH, Schmidt J. 1998. Hydrophobic attachment of Trypanosoma cruzi to the rectal cuticle of Triatoma infestans and its influence on metacyclogenesis - a review. Tokai J Exp Clin Med 23:321–327. 10622629

[42] Dave N, Cetiner U, Arroyo D, Fonbuena J, Tiwari M, Barrera P, Lander N, Anishkin A, Sukharev S, Jimenez V. 2021. A novel mechanosensitive channel controls osmoregulation, differentiation, and infectivity in Trypanosoma cruzi. Elife 10:e67449. doi:10.7554/eLife.67449 34212856 PMC8282336

[43] Jimenez V, Miranda K, Augusto I. 2022. The old and the new about the contractile vacuole of Trypanosoma cruzi*.* J Eukaryot Microbiol 69:e12939. doi:10.1111/jeu.12939 35916682 PMC11178379

[44] Denecke S, Malfara MF, Hodges KR, Holmes NA, Williams AR, Gallagher-Teske JH, Pascarella JM, Daniels AM, Sterk GJ, Leurs R, Ruthel G, Hoang R, Povelones ML, Povelones M. 2024. Adhesion of Crithidia fasciculata promotes a rapid change in developmental fate driven by cAMP signaling. mSphere 9:e0061724. doi:10.1128/msphere.00617-24 39315810 PMC11520290

[45] Wang W, Peng D, Baptista RP, Li Y, Kissinger JC, Tarleton RL. 2021. Strain-specific genome evolution in Trypanosoma cruzi, the agent of chagas disease. PLoS Pathog 17:e1009254. doi:10.1371/journal.ppat.1009254 33508020 PMC7872254

[46] Alvarez-Jarreta J, Amos B, Aurrecoechea C, Bah S, Barba M, Barreto A, Basenko EY, Belnap R, Blevins A, Böhme U, et al.. 2024. VEuPathDB: the eukaryotic pathogen, vector and host bioinformatics resource center in 2023. Nucleic Acids Res 52:D808–D816. doi:10.1093/nar/gkad1003 37953350 PMC10767879

[47] Eisenhaber F, Eisenhaber B, Kubina W, Maurer-Stroh S, Neuberger G, Schneider G, Wildpaner M. 2003. Prediction of lipid posttranslational modifications and localization signals from protein sequences: big-Pi, NMT and PTS1. Nucleic Acids Res 31:3631–3634. doi:10.1093/nar/gkg537 12824382 PMC168944

[48] Paysan-Lafosse T, Blum M, Chuguransky S, Grego T, Pinto BL, Salazar GA, Bileschi ML, Bork P, Bridge A, Colwell L, et al.. 2023. InterPro in 2022. Nucleic Acids Res 51:D418–D427. doi:10.1093/nar/gkac993 36350672 PMC9825450

[49] Goebl M, Yanagida M. 1991. The TPR snap helix: a novel protein repeat motif from mitosis to transcription. Trends Biochem Sci 16:173–177. doi:10.1016/0968-0004(91)90070-C 1882418

[50] Das AK, Cohen PW, Barford D. 1998. The structure of the tetratricopeptide repeats of protein phosphatase 5: implications for TPR-mediated protein-protein interactions. EMBO J 17:1192–1199. doi:10.1093/emboj/17.5.1192 9482716 PMC1170467

[51] Lamb JR, Tugendreich S, Hieter P. 1995. Tetratrico peptide repeat interactions: to TPR or not to TPR? Trends Biochem Sci 20:257–259. doi:10.1016/S0968-0004(00)89037-4 7667876

[52] D’Andrea LD, Regan L. 2003. TPR proteins: the versatile helix. Trends Biochem Sci 28:655–662. doi:10.1016/j.tibs.2003.10.007 14659697

[53] Won MM, Baublis A, Burleigh BA. 2023. Proximity-dependent biotinylation and identification of flagellar proteins in Trypanosoma cruzi. mSphere 8:e0008823. doi:10.1128/msphere.00088-23 37017578 PMC10286712

[54] Contreras VT, Salles JM, Thomas N, Morel CM, Goldenberg S. 1985. In vitro differentiation of Trypanosoma cruzi under chemically defined conditions. Mol Biochem Parasitol 16:315–327. doi:10.1016/0166-6851(85)90073-8 3903496

[55] Boutin JA. 1997. Myristoylation. Cell Signal 9:15–35. doi:10.1016/s0898-6568(96)00100-3 9067626

[56] Gonçalves CS, Ávila AR, de Souza W, Motta MCM, Cavalcanti DP. 2018. Revisiting the Trypanosoma cruzi metacyclogenesis: morphological and ultrastructural analyses during cell differentiation. Parasit Vectors 11:83. doi:10.1186/s13071-018-2664-4 29409544 PMC5801705

[57] Rohloff P, Docampo R. 2008. A contractile vacuole complex is involved in osmoregulation in Trypanosoma cruzi. Exp Parasitol 118:17–24. doi:10.1016/j.exppara.2007.04.013 17574552 PMC2243178

[58] Rohloff P, Rodrigues CO, Docampo R. 2003. Regulatory volume decrease in Trypanosoma cruzi involves amino acid efflux and changes in intracellular calcium. Mol Biochem Parasitol 126:219–230. doi:10.1016/s0166-6851(02)00277-3 12615321

[59] Jones P, Binns D, Chang HY, Fraser M, Li W, McAnulla C, McWilliam H, Maslen J, Mitchell A, Nuka G, Pesseat S, Quinn AF, Sangrador-Vegas A, Scheremetjew M, Yong SY, Lopez R, Hunter S. 2014. InterProScan 5: genome-scale protein function classification. Bioinformatics 30:1236–1240. doi:10.1093/bioinformatics/btu031 24451626 PMC3998142

[60] Naula C, Schaub R, Leech V, Melville S, Seebeck T. 2001. Spontaneous dimerization and leucine-zipper induced activation of the recombinant catalytic domain of a new adenylyl cyclase of Trypanosoma brucei, GRESAG4.4B. Mol Biochem Parasitol 112:19–28. doi:10.1016/s0166-6851(00)00338-8 11166383

[61] Fischer Weinberger R, Bachmaier S, Ober V, Githure GB, Dandugudumula R, Phan IQ, Almoznino M, Polatoglou E, Tsigankov P, Nitzan Koren R, Myler PJ, Boshart M, Zilberstein D. 2024. A divergent protein kinase A regulatory subunit essential for morphogenesis of the human pathogen Leishmania. PLoS Pathog 20:e1012073. doi:10.1371/journal.ppat.1012073 38551993 PMC11006142

[62] Pandey M, Huang Y, Lim TK, Lin Q, He CY. 2020. Flagellar targeting of an arginine kinase requires a conserved lipidated protein intraflagellar transport (LIFT) pathway in Trypanosoma brucei. J Biol Chem 295:11326–11336. doi:10.1074/jbc.RA120.014287 32587088 PMC7415996

[63] Bourguignon SC, de Souza W, Souto-Padrón T. 1998. Localization of lectin-binding sites on the surface of Trypanosoma cruzi grown in chemically defined conditions. Histochem Cell Biol 110:527–534. doi:10.1007/s004180050314 9826132

[64] Shih HW, Alas GCM, Paredez AR. 2023. Encystation stimuli sensing is mediated by adenylate cyclase AC2-dependent cAMP signaling in Giardia*.* Nat Commun 14:7245. doi:10.1038/s41467-023-43028-1 37945557 PMC10636121

[65] Shih H-W, Alas GCM, Paredez AR. 2024. Unraveling the role of cAMP signaling in Giardia: insights into PKA-mediated regulation of encystation and subcellular interactions. mSphere 9:e0072324. doi:10.1128/msphere.00723-24 39475322 PMC11580427

[66] Shaw S, Roditi I. 2023. The sweet and sour sides of trypanosome social motility. Trends Parasitol 39:242–250. doi:10.1016/j.pt.2023.01.001 36732111

[67] Oberholzer M, Morand S, Kunz S, Seebeck T. 2006. A vector series for rapid PCR-mediated C-terminal in situ tagging of Trypanosoma brucei genes. Mol Biochem Parasitol 145:117–120. doi:10.1016/j.molbiopara.2005.09.002 16269191

[68] Boné GJ, Steinert M. 1956. Isotopes incorporated in the nucleic acids of Trypanosoma mega. Nature 178:308–309. doi:10.1038/178308a0 13358724

[69] Chiurillo MA, Lander N, Bertolini MS, Storey M, Vercesi AE, Docampo R. 2017. Different roles of mitochondrial calcium uniporter complex subunits in growth and infectivity of Trypanosoma cruzi. MBio 8:e00574-17. doi:10.1128/mBio.00574-17 28487431 PMC5424207

[70] Chiurillo Miguel A, Ahmed M, González C, Raja A, Lander N. 2023. Gene editing of putative cAMP and Ca^2+^ -regulated proteins using an efficient cloning-free CRISPR/Cas9 system in Trypanosoma cruzi. J Eukaryot Microbiol 70:e12999. doi:10.1111/jeu.12999 37724511 PMC10841170

[71] Lander N, Ulrich PN, Docampo R. 2013. Trypanosoma brucei vacuolar transporter chaperone 4 (TbVtc4) is an acidocalcisome polyphosphate kinase required for in vivo infection. J Biol Chem 288:34205–34216. doi:10.1074/jbc.M113.518993 24114837 PMC3837161

[72] Wormington JD, Gillum C, Meyers AC, Hamer GL, Hamer SA. 2018. Daily activity patterns of movement and refuge use in Triatoma gerstaeckeri and Rhodnius prolixus (Hemiptera: Reduviidae), vectors of the chagas disease parasite. Acta Trop 185:301–306. doi:10.1016/j.actatropica.2018.06.012 29908170

[73] Vieira CB, Praça YR, Bentes KL da S, Santiago PB, Silva SMM, Silva GDS, Motta FN, Bastos IMD, de Santana JM, de Araújo CN. 2018. Triatomines: trypanosomatids, bacteria, and viruses potential vectors? Front Cell Infect Microbiol 8:405. doi:10.3389/fcimb.2018.00405 30505806 PMC6250844

[74] Batista KK da S, Vieira CS, Florentino EB, Caruso KFB, Teixeira PTP, Moraes C da S, Genta FA, de Azambuja P, de Castro DP. 2020. Nitric oxide effects on Rhodnius prolixus’s immune responses, gut microbiota and Trypanosoma cruzi development. J Insect Physiol 126:104100. doi:10.1016/j.jinsphys.2020.104100 32822690

[75] Fellet MR, Lorenzo MG, Elliot SL, Carrasco D, Guarneri AA. 2014. Effects of infection by Trypanosoma cruzi and Trypanosoma rangeli on the reproductive performance of the vector Rhodnius prolixus. PLoS One 9:e105255. doi:10.1371/journal.pone.0105255 25136800 PMC4138117

