# Supplementary material for: TcCARP3 modulates compartmentalized cAMP signals involved in osmoregulation, infection of mammalian cells, and colonization of the triatomine vector in the human pathogen Trypanosoma cruzi
Source: mBio. 2025 May 23;16(6):e00994-25. doi: 10.1128/mbio.00994-25 (PMC12153354; doi:10.1128/mbio.00994-25)
Supplement: Supplemental Figures — Figures S1 to S4. [file mbio.00994-25-s0001.pdf]

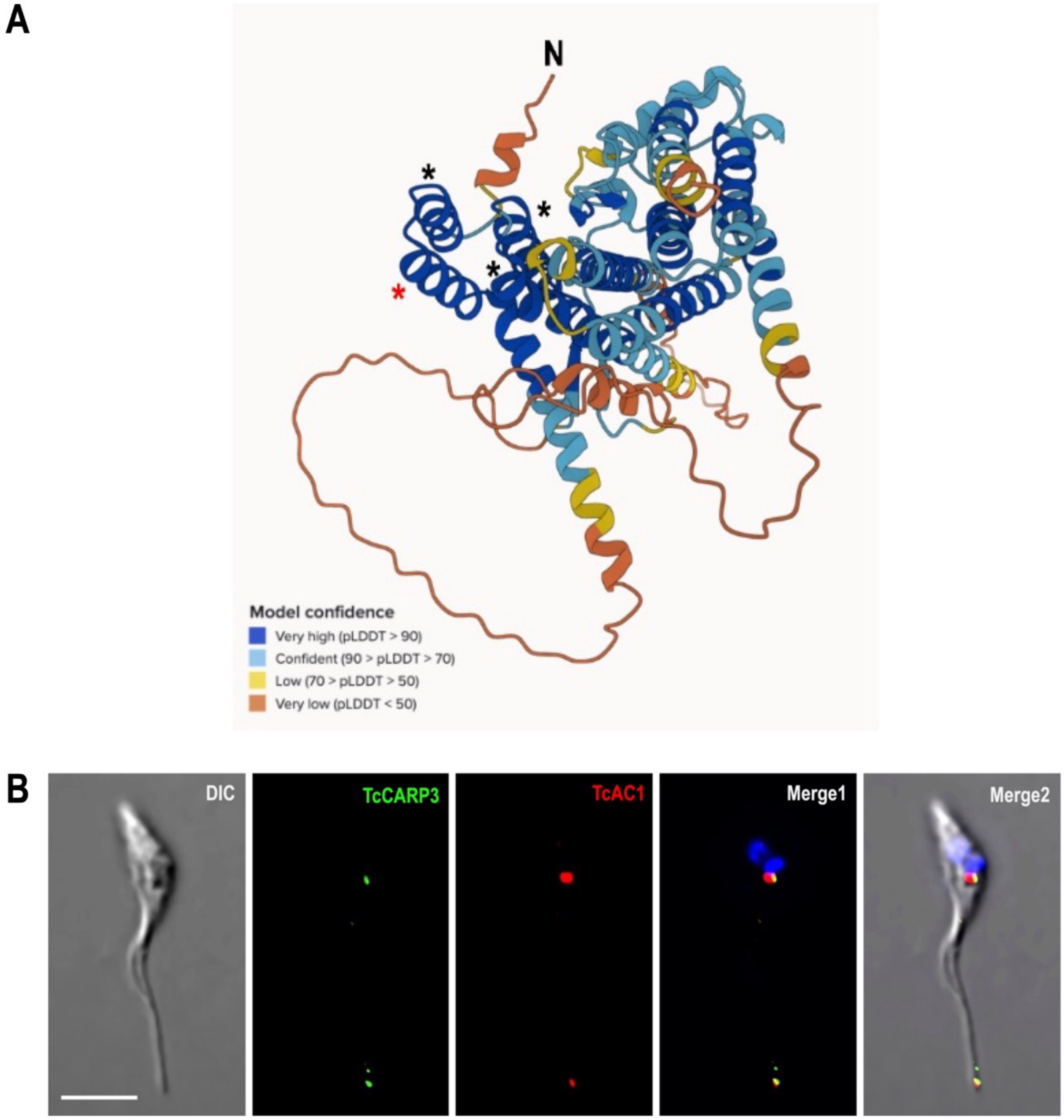

Figure S1. TcCARP3 3D structure and localization under isosmotic conditions. [A] TcCARP3 structure predicted with AlphaFold. Model confidence using per-residue confidence score (pLDDT) is indicated by color-code. Regions below 50 pLDDT may be unstructured in isolation. Asterisks indicate the four alpha helices predicted to constitute the Tetratricopeptide-like helical domain at the N-terminus of TcCARP3. A red asterisk indicates the AC interacting domain as predicted for *T. brucei* CARP3 (17). **[B]** Localization of TcCARP3 and AC1 under normal (isosmotic) conditions. IFAs were performed using TcAC1-3xHA/TcCARP3-3xc-Myc dually tagged cell line under isosmotic conditions in *T. cruzi* epimastigotes. Images from left to right show DIC, TcCARP3 (green), TcAC1 (red), TcCARP3 and TcAC1 merged (yellow) with DAPI (blue), and with DIC. DAPI was used to stain the nucleus and kinetoplast. Scale bars: 5 μm.

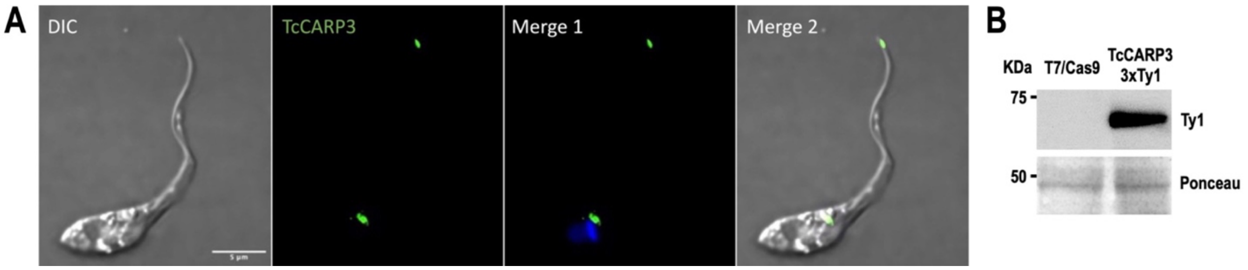

Figure S2. IFA and western blot analysis of endogenously tagged *TcCARP3*-3xTy1. **[A]** Order of IFA panels from left to right is DIC, TcCARP3 (green), TcCARP3 (green) merged with DAPI (blue), and merged with DIC. IFA was performed under hypoosmotic stress to better visualize contractile vacuole localization. Scale bar: 5 µm. **[A]** Western blot analysis of *TcCARP3*-3xTy1 and control (T7/Cas9) cell lines using anti-Ty1 antibodies. Ponceau red staining was used as loading control.

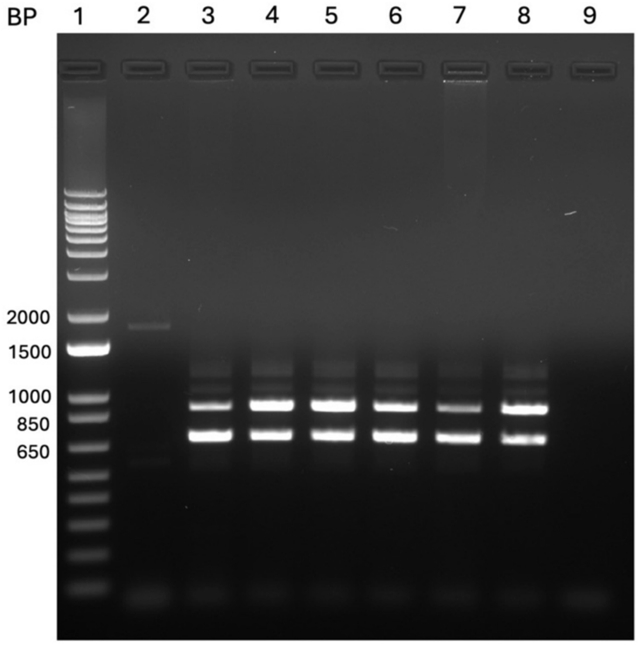

Figure S3. **PCR verification of *TcCARP3*-KO genotype in clonal populations.** Lane 1: 1kb plus ladder, 2: T7/cas9 showing expected WT band of *TcCARP3* at 1.77kb, 3: KO of *TcCARP3* mixed population with bands corresponding to blasticidin (658bp) and puromycin (859bp) resistance markers, 4-8: clones of *TcCARP3* KO, 9: negative control. Further experiments were performed with *TcCARP3*-KO clone G10 (lane 6).

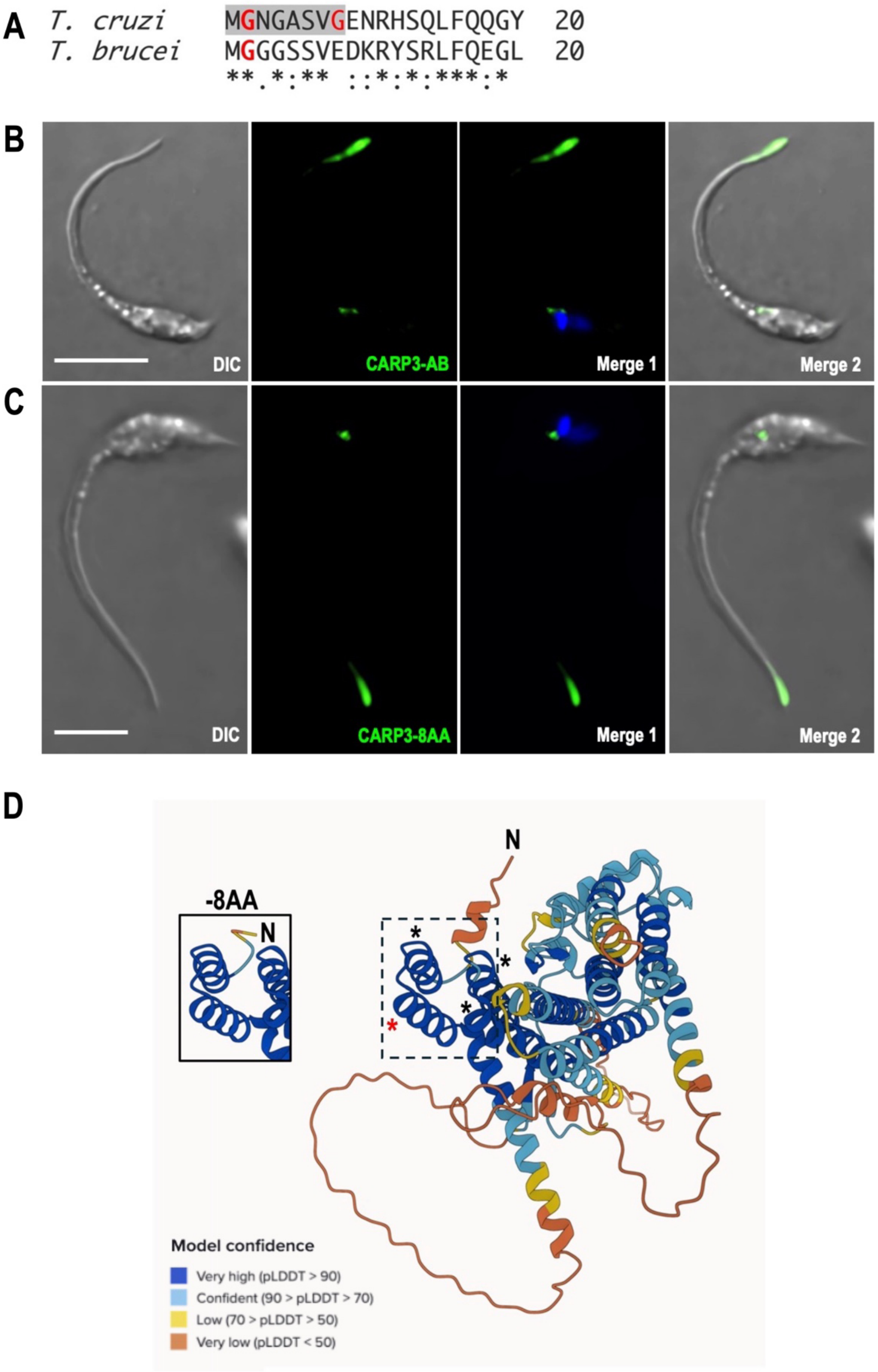
A
T. cruzi MGNGASVGENRHSQLFQQGY 20
T. brucei MGGGSSVEDKRYSRLFQEGL 20
.*: ::*:*:***:*
B
DIC
CARP3-AB
Merge 1
Merge 2
C
DIC
CARP3-8AA
Merge 1
Merge 2
D
-8AA
N
N
Model confidence
Very high (pLDDT > 90)
Confident (90 > pLDDT > 70)
Low (70 > pLDDT > 50)
Very low (pLDDT < 50)

Figure S4. Analysis of TcCARP3 predicted myristoylation signal. [A] Alignment of the first 20 amino acids of CARP3 in *T. cruzi* and *T. brucei*. Putative myristoylation glycines are shown in red. The 8 amino acids removed from TcCARP3 in the truncated mutant *TcCARP3*-8AA are boxed in gray. **[B]** Immunofluorescence analysis of *TcCARP3*-AB. **[C]** IFA showing localization of TcCARP3-8AA in *T. cruzi* epimastigotes. Order of IFA panels from left to right is DIC, TcCARP3 (green), TcCARP3 (green) merged with DAPI (blue), and merged with DIC. IFAs were performed under hypoosmotic stress to better visualize contractile vacuole localization. Scale bars: 5 µm. **[D]** TcCARP3 structure predicted with AlphaFold. The inset at the left side shows the predicted structure of the N-terminal region of TcCARP3-8AA mutant, with no apparent changes in the AC interacting domain (red asterisk). Model confidence using per-residue confidence score (pLDDT) is indicated by color-code. Regions below 50 pLDDT may be unstructured in isolation. Asterisks indicate the four alpha helices predicted to constitute the Tetratricopeptide-like helical domain at the N-terminus of TcCARP3. A red asterisk indicates the AC interacting domain as predicted for *T. brucei* CARP3 (17).
