# Supplementary material for: TcCARP3 modulates compartmentalized cAMP signals involved in osmoregulation, infection of mammalian cells, and colonization of the triatomine vector in the human pathogen Trypanosoma cruzi
Source: mBio. 2025 May 23;16(6):e00994-25. doi: 10.1128/mbio.00994-25 (PMC12153354; doi:10.1128/mbio.00994-25)
Supplement: Table S2 — List of oligonucleotides used in this study. [file mbio.00994-25-s0003.pdf]

Table S2. List of oligonucleotides used in this study

N°	Primer name	Sequence (5' → 3')*
1	FwCARP3_XbaI	AGAA*TCTAGA* ATGGGAAACGGCGCCTCAG
2	RvCARP3_EcoRV	AGAA*GATATC* ATCCTCAAACATCATAAAGAGGG
3	CARP3_sgRNA_KO	GAAATTAATACGACTCACTATAGGTGATGGTGTCTGACCTTGAGGTTTTAGA GCTAGAAATAGC
4	FwCARP3_BSD_PAC	AAACAATATAATCCCCAGGAACAACAAGAAAAGACAAAAAGCCGCGGGAAT TCGATTATG
5	RvCARP3_BSD_PAC	ATTTGCGTACATCATCATCATCATCATCGTCATCGGGCGCGAATTCACTAGT GATTTCAC
6	FwCARP3_KO-CHK	GAGCGCAAACAGTTCAGC
7	RvCARP3_KO-CHK	GTCATGGCGAGTTCTTTTGG
8	RvCARP3_EcoRV	CAGT*GATATC* ATCCTCAAACATCATAAAGAGGGAGC
9	FwCARP3-8NTD_XbaI	ACTG*TCTAGA* ATGGAGAACCGCCACTCACAG
10	CARP3_sgRNA_ctag	GAAATTAATACGACTCACTATAGGCTCCCTCTTTATGATGTTTGGTTTTAGAG CTAGAAATAGC
11	FwCARP3_ctag_23T	GCATCATGCGGTCTGTGATGGAGTCCCGCAAGCGTGGCGGCTCCCTCTTTA TGATGTTTGAAGATGGTACCGGGCCCCCCCTCGAG
12	RvCARP3_ctag_23T	CGAGTTCTTTTGGCTCATGTATCATTTGCGTACATCATCATCATCATCATCGT CATCGGGTGGCGGCCGCTCTAGAACTAGTGGAT
13	Fw2xTy1_XhoI	GCTGGGTACCGGGCCCCCC*CTCGAG* GATACCGTCGACCTCGAG
14	Rv2xTy1_XhoI	GGTCCTGGTTAGTATGGAC*CTCGAG* GCCGGAACTGGCTGAATCAAGGGG
15	FwCARP3_ctag-CHK	AGCCGACGTGCAGTTTGAAC
16	RvCARP3_ctag-CHK	GAGCCAAAAGAACTCGCCATGA
17	G00	AAAAGCACCGACTCGGTGCCACTTTTTCAAGTTGATAACGGACTAGCCTTAT TTTAACTTGCTATTTCTAGCTCAAAAC

*Restriction sites are shown in italics and protospacers are underlined.
